# Gm26550 Modulates Learning and Memory by Increasing IGF1 mRNA Expression and Stability in Nrf2^−/−^ Mice

**DOI:** 10.1002/advs.202524087

**Published:** 2026-09-27

**Authors:** Hongfang Wang, Ziyao Wang, Dongyue Zuo, Zhaowen Su, Bowen Song, Jiamin Gao, Yizhou Zhang, Ruiting Zhao, Linjie Sun, Mengdi Li, Yirui Fan, Dandan Geng, Lei Wang

**Affiliations:** ^1^ Department of Human Anatomy Hebei Medical University Shijiazhuang China; ^2^ Grade 2023 5+3 Clinical Medicine Hebei Medical University Shijiazhuang China; ^3^ Grade 2023 Clinical Medicine Hebei Medical University Shijiazhuang China; ^4^ Hebei Key Laboratory of Neurodegenerative Disease Mechanism Shijiazhuang China; ^5^ Neuroscience Research Center Hebei Medical University Shijiazhuang China; ^6^ Grade 2023 Clinical Medicine Clinical College of Hebei Medical University Shijiazhuang China; ^7^ The Key Laboratory of Neural and Vascular Biology Ministry of Education Hebei Medical University Shijiazhuang China

**Keywords:** IGF1, lncRNA Gm26550, learning and memory, Nrf2

## Abstract

Nrf2 dysfunction is implicated in learning and memory deficits, but the involvement of non‐coding RNAs in Nrf2‐dependent cognitive regulation remains largely unexplored. Here, by analyzing the expression profiles of lncRNAs and mRNAs in the hippocampus of wild‐type (WT) and Nrf2 knockout (Nrf2^−/−^) mice, we identified a decreased expression of lncRNA Gm26550 in Nrf2^−/−^ mice. Overexpression of Gm26550 enhances hippocampal neuronal synaptic plasticity and rescues learning and memory deficits in Nrf2^−/−^ mice. Mechanistically, Nrf2 directly binds the promoter of Gm26550 to drive its transcriptional activation and upregulate its expression. Gm26550 elevates IGF1 abundance via two regulatory pathways to maintain cognitive function: first, it functionally counteracts miR‐26a‐5p‐mediated repression of IGF1; second, it interacts with the KH3/KH4 domain of KHSRP to block KHSRP‐triggered degradation of IGF1 mRNA. Collectively, our results reveal that Gm26550 maintains IGF1 expression and transcript stability through miR‐dependent antagonism and RBP sequestration, thereby governing learning and memory downstream of Nrf2. This work expands the understanding of the downstream molecular network controlled by Nrf2.

## Graphical abstract

The present study shows that Nrf2 directly binds to the Gm26550 promoter, thereby activating Gm26550 transcription and increasing its expression. Mechanistically, Gm26550 promotes IGF1 expression by functionally antagonizing miR‐26a‐5p‐mediated repression and sequestering the RBP KHSRP, thereby enhancing hippocampal neuronal synaptic plasticity and rescuing learning and memory deficits.

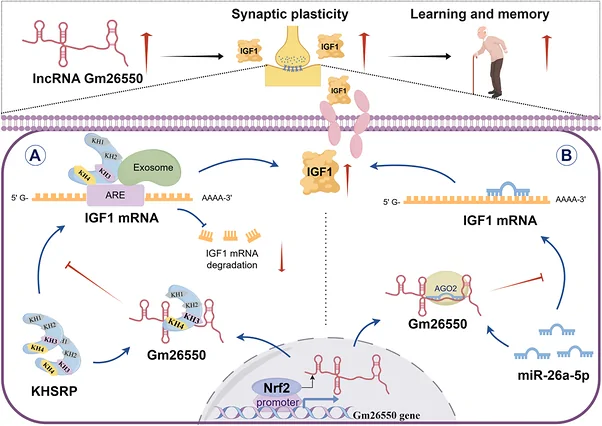

## 1 Introduction

Learning and memory are fundamental higher‐order cognitive functions of the brain and form core components of cognitive ability. Learning is the process by which humans and animals acquire novel information from the external environment. Memory is the set of processes by which the brain encodes, stores, and retrieves acquired information [1, 2]. However, learning and memory impairments are common in neurodegenerative diseases, particularly in Alzheimer's disease (AD) [3] and Parkinson's disease (PD) [4], both of which severely compromise health and quality of life in the elderly population.

Nuclear factor erythroid 2‐related factor 2 (Nrf2/Nfe2l2), a pivotal regulator of the cellular antioxidant defense network, is a key transcription factor that protects neuronal homeostasis and mitigates oxidative stress‐induced neuronal damage [5, 6, 7]. Under physiological conditions, Nrf2 is retained in the cytoplasm through its interaction with Kelch‐like ECH‐associated protein 1 (KEAP1). Upon stimulation by reactive oxygen species (ROS) or ROS mimetics, Nrf2 levels are markedly increased, accompanied by its translocation to the nucleus. There, Nrf2 activates the transcription of antioxidant genes bearing antioxidant response elements (AREs) in their promoter regions, including heme oxygenase‐1 (HO‐1), NAD(P) H quinone dehydrogenase 1 (NQO1), and glutathione S‐transferase π1 (GSTP1) [8, 9]. Accumulating evidence from our group indicates that Nrf2 plays a crucial role in regulating learning and memory [10, 11, 12, 13]. However, the mechanisms by which Nrf2 modulates learning and memory are still not fully understood.

Long noncoding RNAs (lncRNAs) are a class of RNA molecules longer than 200 nucleotides. Through specific interaction modes involving DNA, RNA, or proteins, lncRNAs have been shown to play pivotal roles in a wide range of biological processes and diseases [14]. LncRNAs act as decoys to sequester proteins and microRNAs (miRNAs), as scaffolds that mediate protein–protein interactions, or as guides that orchestrate protein–DNA interactions, thereby regulating gene expression at epigenetic, transcriptional, translational, posttranscriptional, and posttranslational levels [15, 16]. A growing body of evidence indicates that lncRNAs play critical roles in regulating synaptic structure and neuronal function. LncRNA Synage governs cerebellar synaptic structure and function during development through two mechanisms: (i) molecular sponging of miR‐325‐3p to regulate CBLN1 and (ii) scaffolding to mediate the LRP1‐HSP90AA1‐PSD95 synaptic complex assembly [17]. LncRNA Gas5 acts as an activity‐responsive scaffold to mediate cAMP‐dependent synaptic plasticity [18]. It has been reported that nucleolar lncRNA LoNA regulates ribosome biogenesis by dually targeting nucleolin at the 5′‐end and fibrillarin at the 3′‐end; its activity‐dependent downregulation enhances protein translation, synaptic function, and memory [19].

It remains poorly defined whether Nrf2 governs learning and memory via long non‐coding RNAs. In our previous work, we performed whole‐transcriptome microarray profiling to compare lncRNA expression in the hippocampus of WT and Nrf2^−/−^ mice [10]. Subsequent bioinformatic screening pinpointed the human‐conserved lncRNA Gm26550 as a key regulator of cognitive function, which modulates learning and memory by tuning insulin‐like growth factor 1 (IGF1) abundance. Mechanistically, Gm26550 controls IGF1 expression through two axes: first, it functionally antagonizes miR‐26a‐5p to mitigate miR‐26a‐5p‐imposed repression of IGF1; second, it engages the KH3/KH4 domains of KH‐type splicing regulatory protein (KHSRP) to abrogate KHSRP‐mediated degradation of IGF1 mRNA. Together, these two regulatory pathways boost IGF1 levels, strengthen hippocampal synaptic plasticity, and rescue Nrf2 loss‐induced learning and memory deficits.

## 2 Results

### 2.1 LncRNA Gm26550 is Down‐Regulated in the Hippocampus of Nrf2^−/−^ Mice

**FIGURE 1:**
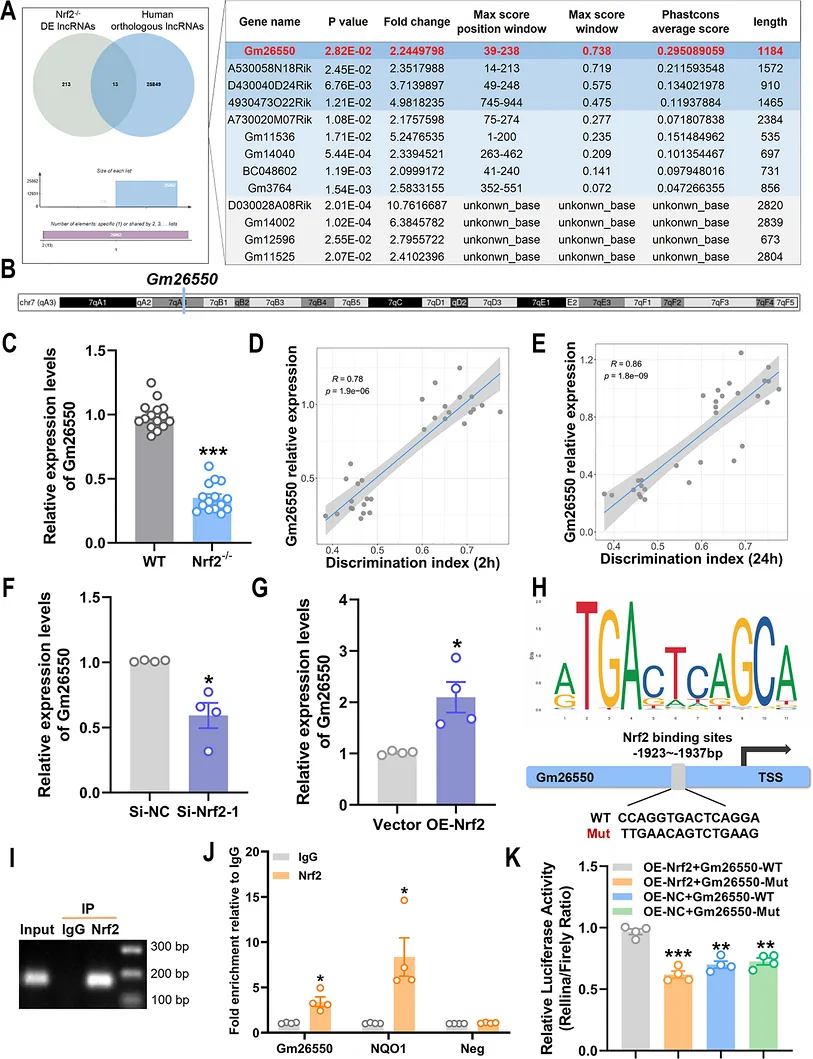
The expression of Gm26550 is down‐regulated in the hippocampus of Nrf2^−/−^ mice. (A) Venn diagram illustrating the overlap between hippocampal differentially expressed lncRNAs in Nrf2^−/−^ mice (vs. WT) and human homologous lncRNAs. The overlap indicates conserved DE‐lncRNAs with potential cross‐species functional relevance. (B) The chromosomal position of the mouse Gm26550 was retrieved from the UCSC Genome Browser database. (C) qRT‐PCR analysis of Gm26550 expression in the hippocampal tissue of Nrf2^−/−^ mice. Gm26550 expression levels were normalized to those in WT mice. (n = 15). (D,E) Correlation analysis between Gm26550 expression and DI in WT and Nrf2^−/−^ mice. (n = 15). (F,G) qRT‐PCR analysis of Gm26550 expression in HT22 cells following Nrf2 knockdown or overexpression. (n = 4). (H) Schematic illustration of the wild‐type (WT) and mutant (Mut) sequences of the Gm26550 motifs. (I,J) ChIP‐qPCR analysis of Nrf2 occupancy at the Gm26550 promoter, the NQO1 promoter (positive control), and an intergenic non‐binding region (negative control, Neg) in HT22 cells. Immunoprecipitation was performed using an anti‐Nrf2 antibody or control rabbit IgG. (n = 4). (K) Dual‐luciferase reporter assays were performed to verify that Nrf2 promotes Gm26550 expression by directly binding to its promoter region. (n = 4). Data are presented as the mean ± SEM. *p < 0.05, **p < 0.01, and ***p < 0.001 by two‐tailed *t‐*test (C, F, G, and J) or Pearson correlation test (D and E), or one‐way ANOVA followed by Bonferroni's post hoc test (K).

To investigate the potential role of Nrf2 in learning and memory, we first generated Nrf2^−/−^mice. The genotype of Nrf2^−/−^ mice was identified by PCR, and the knockout efficiency was confirmed by qRT‐PCR (Figure S1A,B). Nrf2^−/−^ mice appeared grossly normal and showed no significant difference in body weight compared with WT littermates. Subsequently, we confirmed the presence of learning and memory impairments in Nrf2^−/−^ mice using behavioral tests (Figure S1C–J). To explore the underlying molecular mechanisms, we analyzed the lncRNA expression profile in the hippocampus of Nrf2^−/−^ and WT mice using previously published microarray data (GSE122422) [10]. Differentially expressed (DE) lncRNAs were filtered using the criteria of fold change (FC) > 2.0 and *p* < 0.05 (Tables S1–S4). We further focused on the significantly downregulated lncRNAs in hippocampal tissue and identified 13 highly conserved candidate lncRNAs by comparing them with the list of human homologous lncRNAs (Figure 1A). Among these candidates, the lncRNA Gm26550, located on chromosome 7qA3, exhibited the highest scores for both human sequence conservation and ultra‐conserved regions (Figure 1B). Through the UCSC Genomics Institute Bioinformatics website (http://genome.ucsc.edu/), we identified that the genomic region flanking the Gm26550 locus exhibits interspecies conservation (Figure S1K,L). qRT‐PCR assays revealed a significant decrease in Gm26550 expression in the hippocampal region of Nrf2^−/−^ mice (Figure 1C). Moreover, Pearson's correlation analysis showed that hippocampal Gm26550 expression positively correlates with the discrimination index (DI), an essential metric of learning and memory derived from the NOR test (Figure 1D,E).

To investigate whether Nrf2 directly regulates Gm26550 transcription, we manipulated Nrf2 expression in HT22 hippocampal neuronal cells and examined the corresponding changes in Gm26550 levels. Knockdown and overexpression of Nrf2 were first validated at both mRNA and protein levels (Figure S1M–R). Gm26550 expression was significantly reduced upon Nrf2 knockdown and markedly increased upon Nrf2 overexpression (Figure 1F,G), indicating that Nrf2 positively regulates Gm26550 expression in this cellular context. Then we searched for potential Nrf2‐binding sites in the Gm26550 promoter region using the JASPAR database (http://jaspar.genereg.net/) and identified a putative Nrf2‐responsive motif (Figure 1H and Figure S1S). Chromatin immunoprecipitation (ChIP) in HT22 cells confirmed that Nrf2 directly associates with this motif, located at positions 1923–1937 bp relative to the transcriptional start site (Figure 1I,J). Consistently, dual‐luciferase reporter assays showed that Nrf2 overexpression significantly enhanced the activity of the Gm26550‐WT reporter, whereas no effect was observed for the Gm26550‐Mut reporter (Figure 1K). Collectively, these findings demonstrate that Nrf2 can promote Gm26550 transcription by binding to its promoter region in HT22 cells. We further observed that Gm26550 expression was significantly downregulated in the hippocampus of 5×FAD mice and, importantly, also in hippocampal tissues from AD patients (Figure S2A, B), extending the pathophysiological relevance of Gm26550 downregulation to clinically relevant contexts. Collectively, these findings position Gm26550 as a potential downstream effector of Nrf2 in the regulation of learning and memory.

### 2.2 Overexpression of Gm26550 in the Hippocampus Rescues Learning and Memory Impairments in Nrf2^−/−^ Mice

**FIGURE 2:**
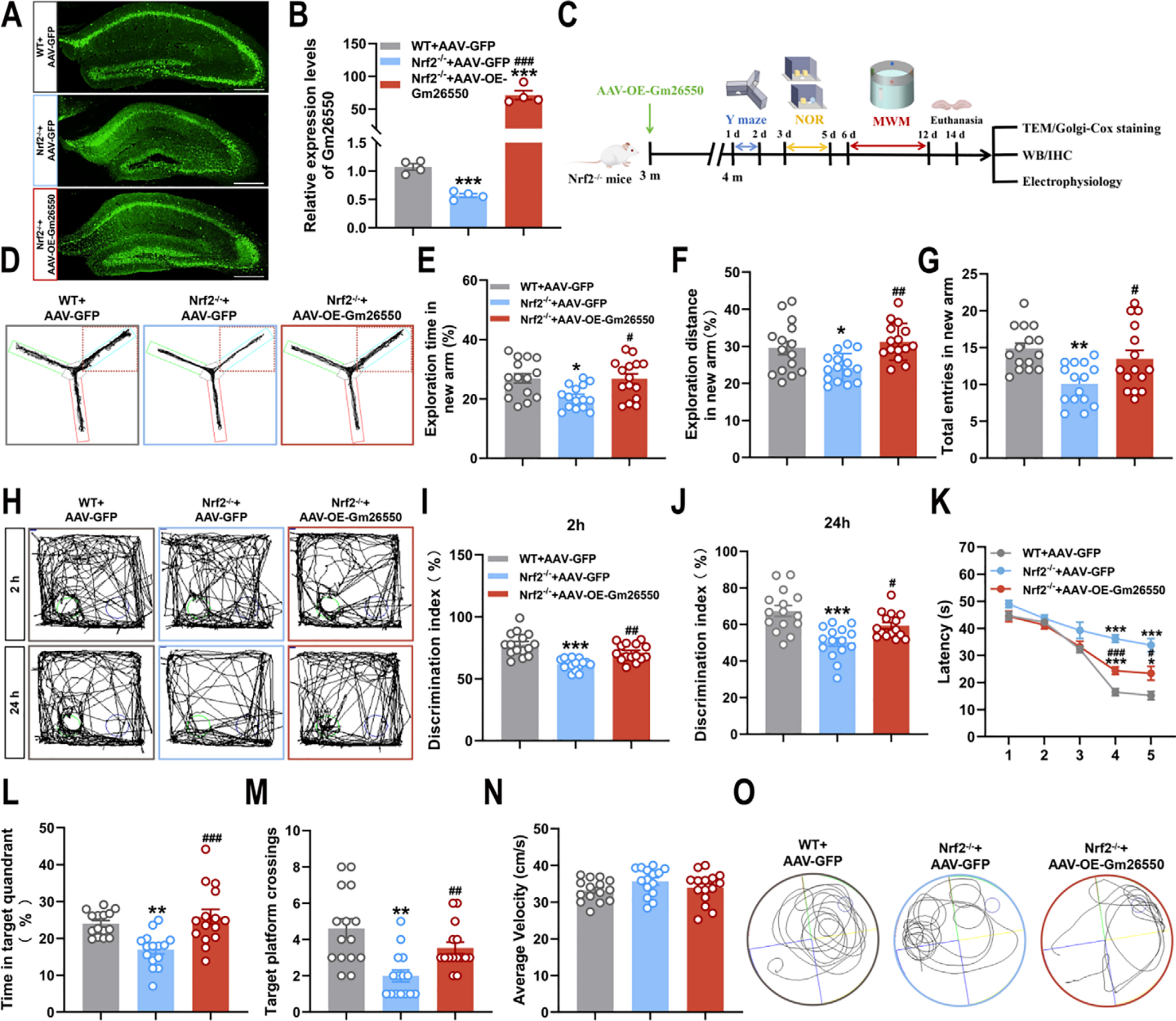
Overexpression of Gm26550 rescues memory impairments in Nrf2^−/−^ mice. (A) Representative fluorescence images showing green fluorescent protein (GFP) expression in the hippocampus of WT and Nrf2^−/−^ mice after stereotaxic injection. (Scale bars = 500 µm). (B) qRT‐PCR analysis of Gm26550 expression in the hippocampus after viral injection. (n = 4). (C) Schematic diagram of the experimental timeline. (D–G) Y‐maze test evaluating the percentage of time spent in the new arm, the percentage of distance traveled in the new arm, and the number of entries into the new arm. (n = 15). (H–J) NOR test performed at 2 h and 24 h to assess preference for familiar versus novel objects. (n = 15). (K‐O) MWM task to assess behavioral performance. (n = 15). (K) Escape latency (average latency) from Days 2 to 6. (L) Time spent in the target quadrant on Day 7. (M) Number of platform crossings on Day 7. (N) Average swim speed during the probe trial. (O) Representative swimming trajectories on Day 7 (n = 15). Data are presented as the mean ± SEM. *p < 0.05, **p < 0.01, ***p < 0.001, ^#^ *p* < 0.05, ^##^ *p* < 0.01, ^###^ *p* < 0.001 by one‐way ANOVA (B, E, F, G, I, J, L, M, and N) or two‐way ANOVA (K) followed by Bonferroni's post hoc test.

To investigate the role of Gm26550 in regulating learning and memory, we constructed recombinant adeno‐associated viral (AAV) vectors: AAV‐GFP (control) and AAV‐OE‐Gm26550 (overexpression). These vectors were bilaterally microinjected into the hippocampus of 3‐month‐old Nrf2^−/−^ mice to locally upregulate Gm26550 expression in this brain region. Four weeks after injection, the transfection efficiency of AAV‐Gm26550 was validated by qRT‐PCR (Figure 2A,B). Subsequently, all mice were subjected to a battery of behavioral tests to evaluate their learning and memory capacity, including the Y‐maze, NOR, and MWM tests (Figure 2C). In the Y maze test, the Nrf2^−/−^ + AAV‐OE‐Gm26550 group showed a significantly higher percentage of time spent in the new arm, the percentage of distance traveled in the new arm, and the number of entries into the new arm compared with the Nrf2^−/−^ + AAV‐GFP group (Figure 2D–G). In the NOR test, the WT + AAV‐GFP and Nrf2^−/−^ + AAV‐OE‐Gm26550 groups exhibited a significantly higher discrimination index than the Nrf2^−/−^ + AAV‐GFP group (Figure 2H–J). In addition, the MWM test showed that the hippocampal overexpression of Gm26550 shortened escape latency (Figure 2K). Compared with the Nrf2^−/−^ + AAV‐GFP group, the Nrf2^−/−^ + AAV‐OE‐Gm26550 mice also spent more time in the target quadrant (Figure 2L) and exhibited an increased number of platform crossings (Figure 2M,O). There were no significant differences in mean swim speed among the groups (Figure 2N). Taken together, these findings indicate that hippocampal overexpression of Gm26550 can effectively ameliorate learning and memory impairments in Nrf2^−/−^ mice.

**FIGURE 3:**
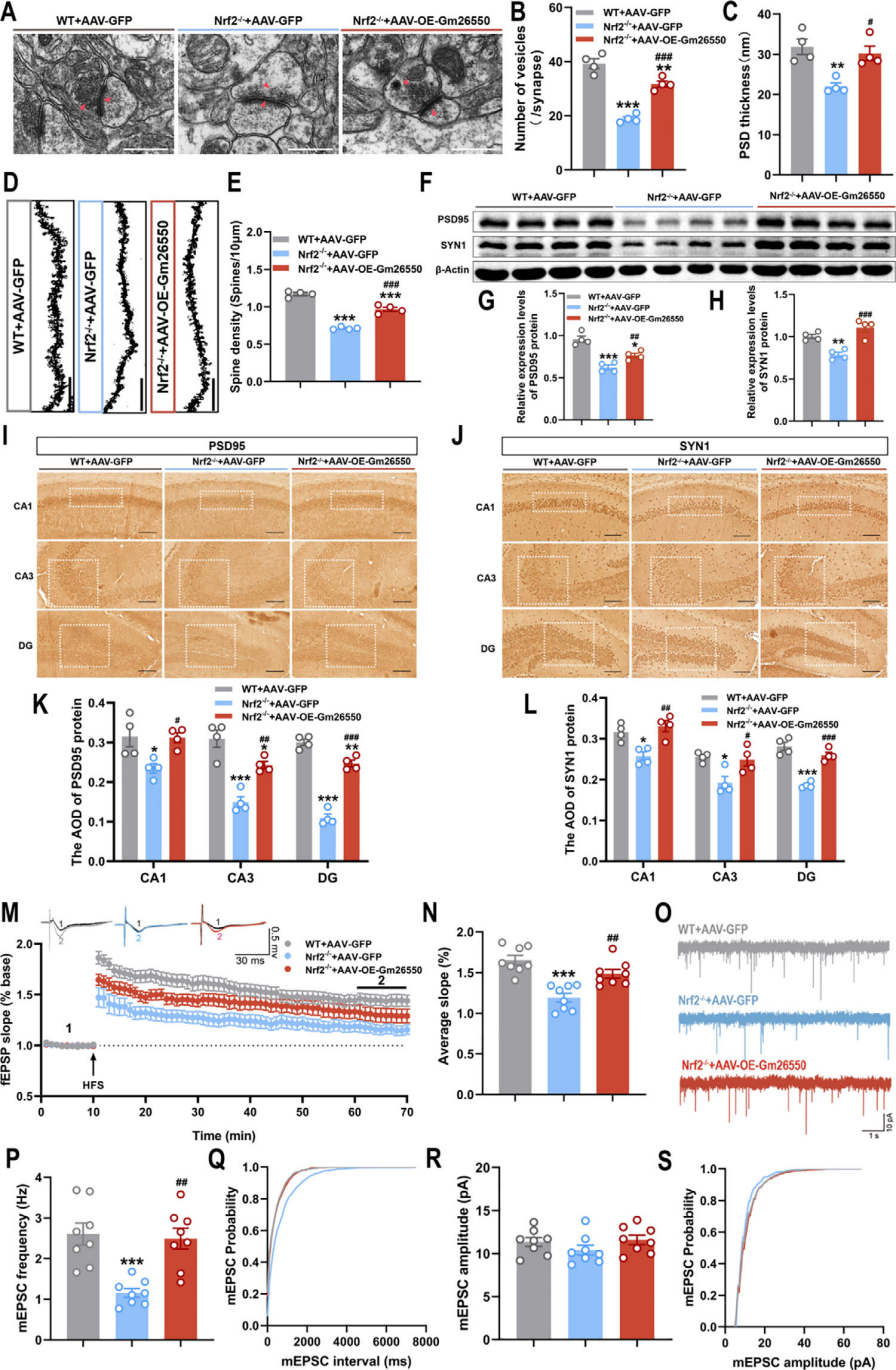
Overexpression of Gm26550 rescues synaptic disorder in Nrf2^−/−^ mice. (A) Representative TEM images of the hippocampal CA1 in the three experimental groups. (n = 4; Scale bars = 1 µm). (B,C) Quantification of TEM observations showing the number of synaptic vesicles (B) and the thickness of the PSD (C). (n = 4). (D) Golgi‐Cox staining shows spine density in the CA1 region of the hippocampus. (Scale bars = 10 µm). (E) Quantification of spine density. (n = 20 neurons from 4 mice). (F) Representative Western blot images of PSD95 and SYN1 expression in the hippocampus. (n = 4). (G‐H) Quantification of PSD95 and SYN1 expression. (I–L) Representative images and quantification of SYN1 and PSD95 in the hippocampus by immunohistochemical staining. (n  =  4, Scale bars  =  50 µm). (M) High‐frequency stimulation (HFS)‐induced LTP was recorded in the Schaffer collateral‐CA1 pathway in mice from the three experimental groups. The arrow indicates the time point of LTP induction by HFS. (N) Quantification of the average fEPSP slope at 60 min. (n = 8 slices from 3 mice). (O) Representative mEPSCs traces of mEPSCs from hippocampal CA1 pyramidal neurons. (P,Q) Quantification of mEPSC frequency. (R,S) Quantification of mEPSC amplitude. (n = 8 cells from 3 mice). Data are presented as the mean ± SEM. *p < 0.05, **p < 0.01, ***p < 0.001, ^#^ *p* < 0.05, ^##^ *p* < 0.01, and ^###^ *p* < 0.001 by one‐way ANOVA followed by Bonferroni's post hoc test (B, C, E, G, H, K, L, N, P, and R).

Synapses, the specialized junctions between adjacent neurons, serve as the primary sites for neural information transmission and provide the structural and functional basis for learning, memory acquisition, consolidation, and retrieval [20]. Transmission electron microscopy (TEM) analysis revealed that Gm26550 overexpression significantly increased two key structural parameters of synapses in the hippocampal CA1 region, including the number of synaptic vesicles (Figure 3A,B) and the thickness of the postsynaptic density (PSD) (Figure 3A,C). Golgi‐Cox staining analysis further demonstrated that Gm26550 overexpression significantly increased the density of dendritic spines in the hippocampus (Figure 3D,E). Consistently, Western blot analysis and immunohistochemical (IHC) staining showed that AAV‐Gm26550 injection markedly reversed the downregulation of synaptic plasticity‐related proteins, including PSD95 and synapsin 1 (SYN1), in the hippocampus of Nrf2^−/−^ mice (Figure 3F–L). Furthermore, we assessed hippocampal long‐term potentiation (LTP) induced by high‐frequency stimulation in the CA3‐CA1 neural circuit. Compared with the WT + AAV‐GFP group, the Nrf2^−/−^ + AAV‐GFP mice exhibited a significant reduction in LTP magnitude; notably, this deficit was rescued in the Nrf2^−/−^ + AAV‐OE‐Gm26550 group (Figure 3M,N). We also recorded miniature excitatory postsynaptic potentials (mEPSPs) from hippocampal neurons in acute brain slices. Compared with WT + AAV‐GFP group, neurons from the Nrf2^−/−^ + AAV‐GFP mice showed a significant decrease in mEPSP frequency, which was reversed by Gm26550 expression, restoring mEPSP frequency to the level of WT + AAV‐GFP (Figure 3O–S). Consistently, loss‐of‐function experiments in HT22 cells further confirmed that siRNA‐mediated knockdown of Gm26550 significantly reduced the protein levels of SYN1 and PSD95 (Figure S3A–C), underscoring the essential role of Gm26550 in maintaining synaptic protein expression. Taken together, these results demonstrate that Gm26550 promotes hippocampal synaptic plasticity in Nrf2^−/−^ mice.

### 2.3 Gm26550 Regulates the Stability and Expression of IGF1 mRNA

**FIGURE 4:**
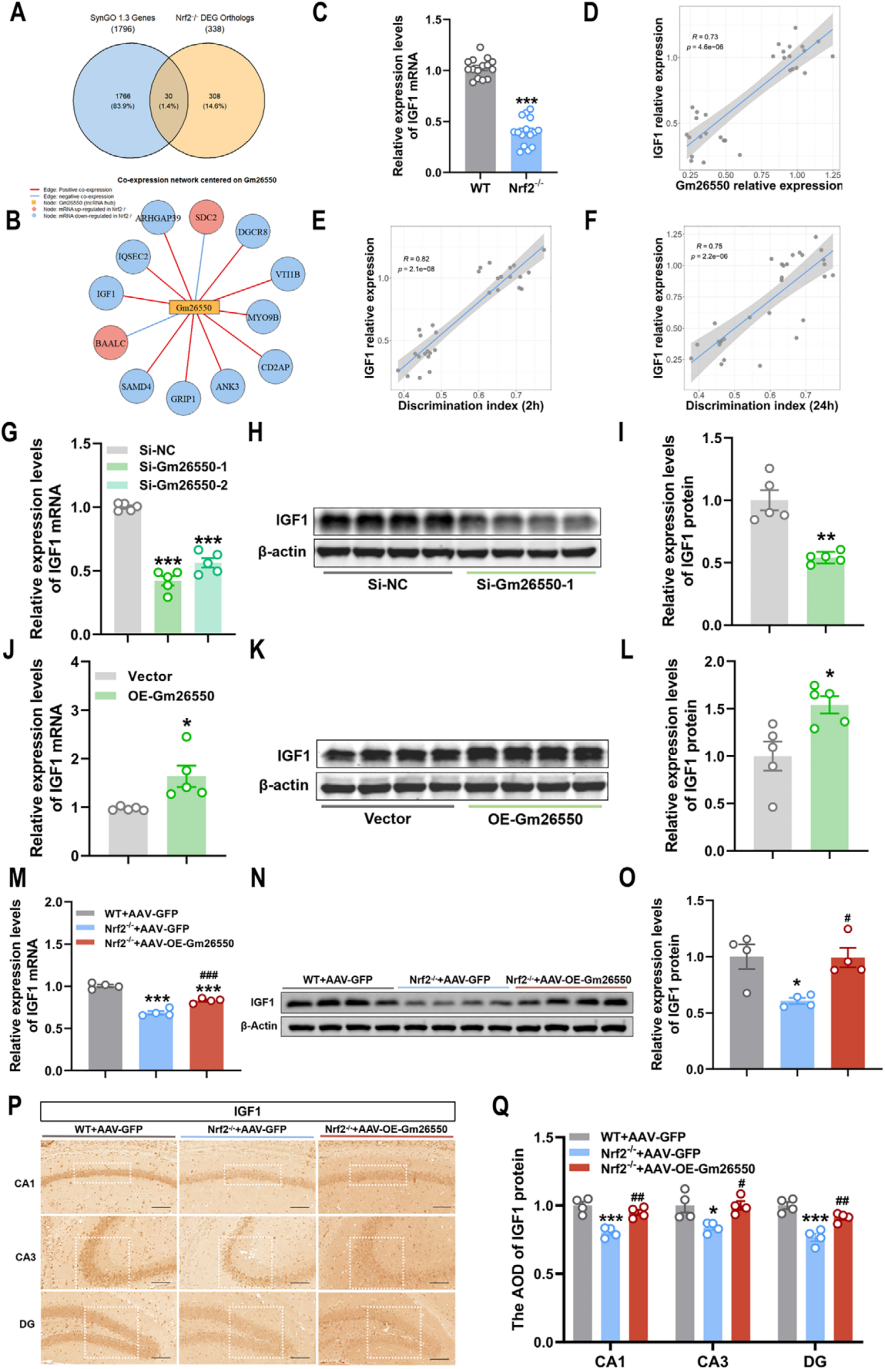
Gm26550 promotes the expression of IGF1. (A) Venn diagram showing the overlap between SynGO 1.3 synaptic genes (1796 total) and Nrf2^−/−^ DEGs orthologs (338 total). A total of 30 intersecting synaptic DEGs were identified for subsequent co‐expression analysis. (B) Co‐expression network centered on the hub lncRNA Gm26550 constructed from the 12 overlapping synaptic DEGs. (C) qRT‐PCR analysis of IGF1 mRNA levels in the hippocampus of Nrf2^−/−^ mice. IGF1 mRNA levels were normalized to those in WT mice. (n = 15). (D) Correlation between Gm26550 and IGF1 in the hippocampus of WT and Nrf2^−/−^ mice. (n = 15). (E,F) Correlation between hippocampal IGF1 levels and the DI at 2 and 24 h in the NOR test. (n = 15). (G–L) qRT‐PCR and Western blot analyses of IGF1 expression in HT22 cells following Gm26550 knockdown or overexpression. (n = 5). (M–O) qRT‐PCR and Western blot analyses of IGF1 expression in the hippocampus across the three mouse groups. (n = 4). (P,Q) Representative images and quantification of IGF1 immunoreactivity in the hippocampus across the three mouse groups. (n  =  4, Scale bars  =  50 µm). Data are presented as the mean ± SEM. *p < 0.05, **p < 0.01, ***p < 0.001, ^#^ *p* < 0.05, ^##^ *p* < 0.01, and ^###^ *p* < 0.001 by two‐tailed *t*‐test (C, I, J, and L) or Pearson correlation test (D, E, and F) or one‐way ANOVA followed by Bonferroni's post hoc test (G, M, O, and Q).

Given the critical role of Gm26550 in modulating synaptic plasticity and memory, we sought to elucidate its underlying molecular mechanisms. To this end, we interrogated hippocampal transcriptomic profiles from the public microarray dataset GSE122422, derived from the hippocampal tissues of Nrf2^−/−^ and WT mice [10]. Applying a threshold of FC > 1.5 and *p* < 0.05, we identified 401 DEGs (Tables S5–S8). Upon mapping these genes to their human orthologs, we intersected the resulting 338 unique identifiers with the SynGO database, prioritizing 30 high‐confidence synaptic candidates for further analysis (Figure 4A, Figure S4A, and Tables S9 and S10). To explore potential downstream targets, we performed a preliminary co‐expression analysis in our experimental cohort. Although the small sample size limits the power of this correlation analysis, it allowed us to identify a subset of genes exhibiting strong co‐expression patterns with Gm26550 (|*r*| ≥ 0.8, *p* < 0.05; Figure 4B, Figure S4B, and Tables S11 and S12). Among these candidates, IGF1 exhibited the most striking coordinated expression trend with Gm26550 (Figure S4C–F). qRT‐PCR and Pearson's correlation analysis further verified that IGF1 exhibited the strongest positive correlation with Gm26550 at the mRNA level (Figure 4C,D). IGF1 also showed a significant association with the DI in the NOR test (Figure 4E,F), making it a promising candidate for subsequent functional validation.

To validate these bioinformatic findings, we evaluated the regulatory relationship between Gm26550 and IGF1 in vitro. In HT22 cells, Gm26550 knockdown significantly reduced IGF1 mRNA and protein levels, whereas its overexpression elicited a marked increase in IGF1 expression (Figure 4G–L and Figure S5A,B). Consistent with these in vitro results, IGF1 was significantly downregulated in the hippocampi of Nrf2^−/−^ mice compared with wild‐type controls, as confirmed by qRT‐PCR, Western blot, and immunohistochemical analyses (Figure 4M–Q). Crucially, the IGF1 deficiency in the Nrf2^−/−^ hippocampus was effectively reversed by Gm26550 overexpression, identifying IGF1 as a vital downstream effector in Gm26550‐mediated synaptic regulation.

### 2.4 Gm26550 Functionally Antagonizes miR‐26a‐5p to Elevate IGF1 Expression

**FIGURE 5:**
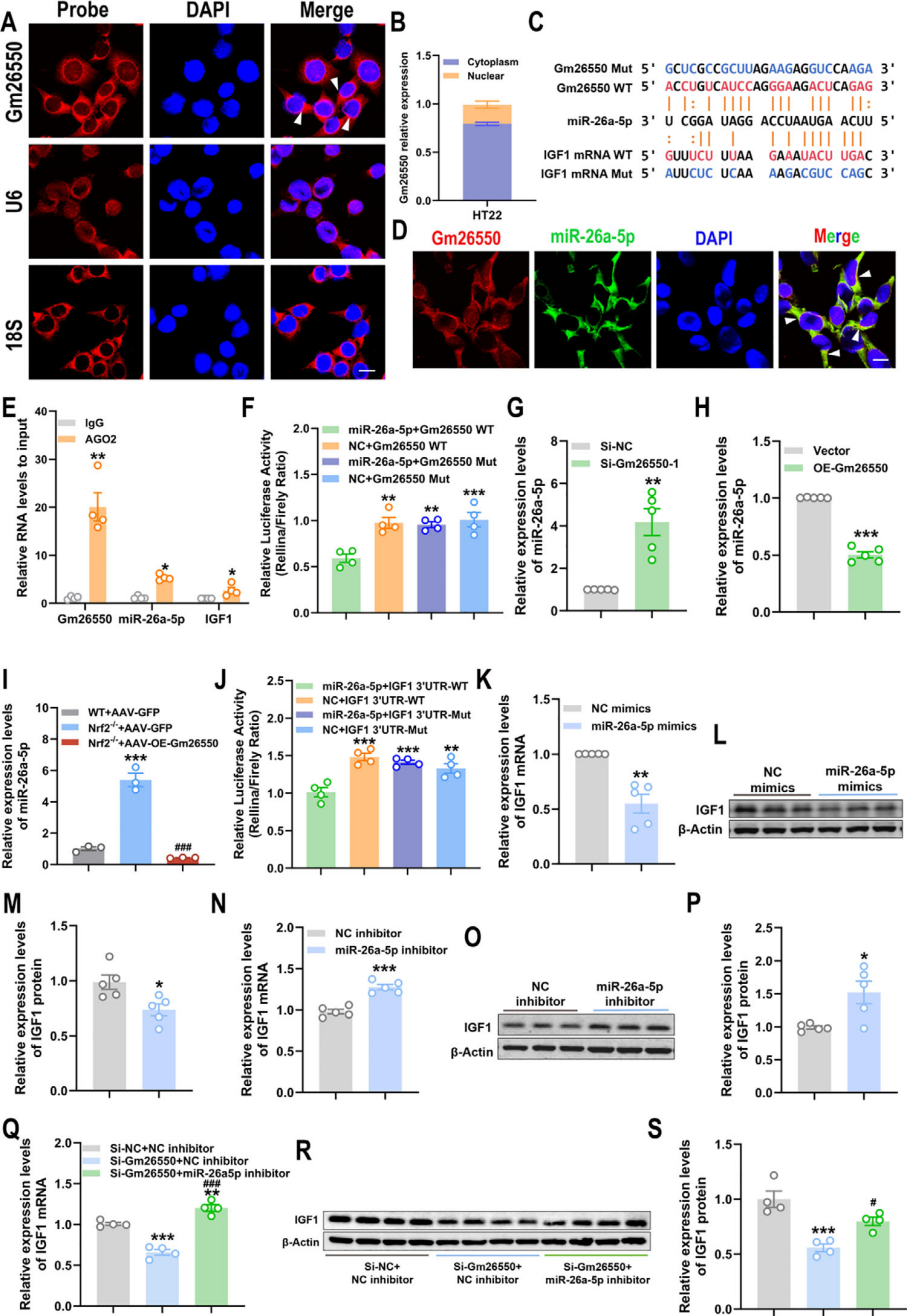
Gm26550 regulates IGF1 expression by sponging miR‐26a‐5p. (A) FISH showed the localization of Gm26550 in HT22 cells. The Gm26550 probe was labeled with Cy3 (red), and nuclei were counterstained with DAPI (blue). (Scale bars = 50 µm). (n = 3). (B) FISH statistical data for Gm26550 subcellular localization. (n = 3). (C) Schematic illustration of the Gm26550‐WT, Gm26550‐Mut, IGF1‐WT, and IGF1‐Mut luciferase reporter constructs. The mutation sequence is shown in red. (D) FISH showed the cytoplasmic co‐localization of Gm26550 and miR‐26a‐5p. (Scale bars = 50 µm). (n = 3). (E) RIP assays were performed in HT22 cells using an anti‐AGO2 antibody. The enrichment of Gm26550, miR‐26a‐5p, and IGF1 mRNA in the immunoprecipitated complexes was determined by qRT‐PCR. (n = 4). (F) A dual luciferase reporter assay was performed to verify the interaction between Gm26550 and miR‐26a‐5p. (n = 4). (G,H) qRT‐PCR was used to detect miR‐26a‐5p levels in HT22 cells following Gm26550 knockdown or overexpression. (n = 5). (I) qRT‐PCR analysis was performed to detect miR‐26a‐5p expression in Nrf2^−/−^ mice after AAV‐OE‐Gm26550. (n = 3). (J) A dual luciferase reporter assay was performed to prove the direct targeting of IGF1 mRNA by miR‐26a‐5p. (n = 4). (K–P) After transfection with miR‐26a‐5p mimics and inhibitor, IGF1 expression in HT22 cells was examined by qRT‐PCR and Western blot. (n = 5). (Q–S) qRT‐PCR and Western blot were used to detect IGF1 expression after co‐transfection of Si‐Gm26550 and a miR‐26a‐5p inhibitor in HT22 cells. (n = 4). Data are presented as the mean ± SEM. *p < 0.05, **p < 0.01, ***p < 0.001, ^#^ *p* < 0.05, and ^###^ *p* < 0.001 by two‐tailed *t*‐test (E, G, H, K, M, N, and P) or one‐way ANOVA followed by Bonferroni's post hoc test (F, I, J, Q, and S).

The functions of lncRNAs are largely associated with their intracellular localization. We therefore performed FISH assays. The results showed that Gm26550 was mainly localized in the cytoplasm (Figure 5A,B). To explore the potential regulatory relationship between Gm26550 and IGF1, we conducted an integrative cross‐analysis using four miRNA target prediction databases (miRanda, TargetScan, miRcode, and RNAhybrid). miR‐26a‐5p was the only miRNA identified as a common intersection among the four databases (Figure S6A). Bioinformatics analysis further suggested that both Gm26550 and IGF1 contain putative microRNA response elements (MREs) for miR‐26a‐5p (Figure 5C). Next, to assess subcellular colocalization, we co‐stained HT22 cells for Gm26550 and miR‐26a‐5p. The two molecules showed predominant cytoplasmic colocalization (Figure 5D). Then, we performed RIP using an anti‐AGO2 antibody and demonstrated successful enrichment of Gm26550, miR‐26a‐5p and IGF1 mRNA (Figure 5E and Figure S6B). Additionally, RNA pull‐down assays using a biotin‐labeled Gm26550 probe confirmed its direct association with AGO2 protein (Figure S6C).

Subsequently, a dual‐luciferase reporter assay showed that miR‐26a‐5p mimics significantly attenuated luciferase activity of the WT Gm26550 reporter, whereas no difference was observed for the Mut Gm26550 reporter (Figure 5F). qRT‐PCR analysis further revealed that Gm26550 overexpression significantly decreased, whereas Gm26550 knockdown markedly increased, the expression of miR‐26a‐5p in HT22 cells (Figure 5G,H). Moreover, Gm26550 overexpression significantly reduced the miR‐26a‐5p expression in Nrf2^−/−^ mice (Figure 5I).

To validate whether miR‐26a‐5p directly targets IGF1 mRNA, we mutated the predicted miRNA binding site in the 3′UTR of IGF1 and performed dual‐luciferase reporter assays. The results showed that miR‐26a‐5p could bind to the IGF1 3′UTR (Figure 5J). After transfection with miR‐26a‐5p mimics, both IGF1 mRNA and protein levels were downregulated (Figure 5K–M). Conversely, transfection with a miR‐26a‐5p inhibitor increased IGF1 mRNA and protein expression (Figure 5N–P). Furthermore, in HT22 cells, the miR‐26a‐5p inhibitor abrogated the downregulation of IGF1 induced by Gm26550 knockdown (Figure 5Q–S). Taken together, these cellular and molecular assays demonstrate that Gm26550 counteracts miR‐26a‐5p‐mediated repression of IGF1, thereby sustaining IGF1 expression.

### 2.5 Gm26550 Stabilizes IGF1 mRNA by Interacting With KHSRP

**FIGURE 6:**
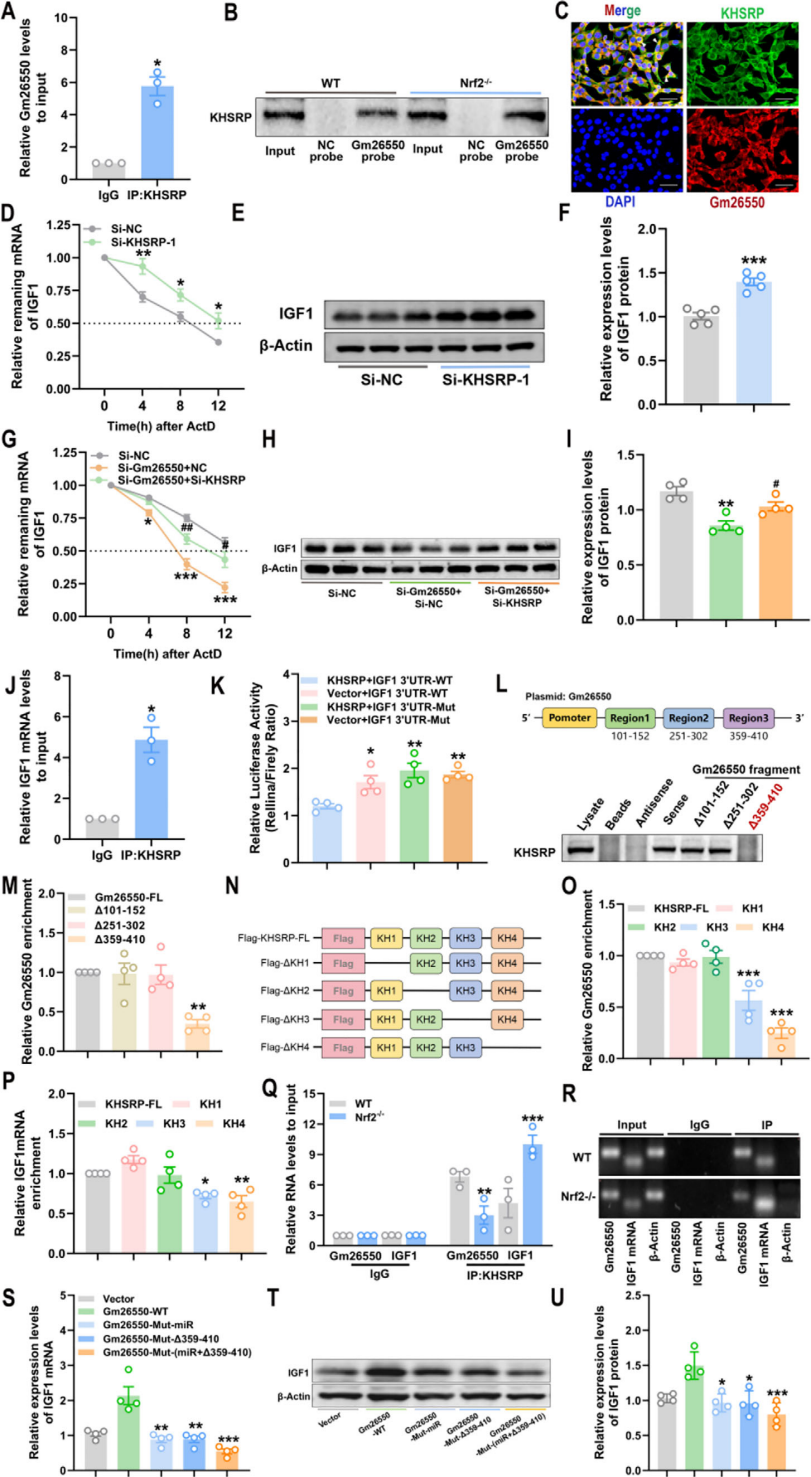
Gm26550 interacts with KHSRP to regulate IGF1 expression and mRNA stability. (A) RIP–qRT‐PCR analysis was performed using hippocampal tissues from WT mice to detect the enrichment of Gm26550 in KHSRP immunoprecipitated complexes. (n = 3). (B) Gm26550 RNA pull‐down was performed using hippocampal tissues from WT mice, followed by Western blot to detect KHSRP enrichment in the pulled‐down complexes. (n = 3). (C) FISH combined with immunofluorescence staining showed colocalization of Gm26550 and KHSRP in the cytoplasm. (n = 3; Scale bars = 50 µm). (D) qRT‐PCR was used to measure IGF1 mRNA levels over time in HT22 cells following KHSRP knockdown. (n = 3). (E,F) IGF1 expression in HT22 cells was analyzed by Western blot following KHSRP knockdown. (n = 5). (G) qRT‐PCR was used to assess IGF1 mRNA levels over time in HT22 cells after cotransfection with si‐Gm26550 and si‐KHSRP. (n = 3). (H–I) Western blot was used to analyze IGF1 expression after cotransfection with si‐Gm26550 and si‐KHSRP in HT22 cells. (n = 4). (J) RIP assays were performed using hippocampal tissues from WT mice with an anti‐KHSRP antibody, and IGF1 mRNA enrichment was determined by qRT‐PCR. (n = 3). (K) A dual luciferase reporter assay shows the activity of the IGF1 3′UTR. (n = 4). (L) RNA pull‐down assay reveals three putative interaction sites between Gm26550 and KHSRP. (M) qRT‐PCR analysis demonstrated that the 359–410 nt region of Gm26550 is required for its association with KHSRP in HEK293 cells. (n = 4). (N) Schematic representation of full‐length KHSRP and truncated constructs. (O) qRT‐PCR showed that the KH3‐4 domains of KHSRP are required for interaction with Gm26550 in HEK293 cells. (n = 4). (P) qRT‐PCR showed that the KH3 and KH4 domains are required for interaction with IGF1 mRNA in HEK293 cells. (n = 4). (Q) RIP‐qRT‐PCR analysis was performed in hippocampal tissues from Nrf2^−/−^ mice to determine Gm26550 and IGF1 mRNA enrichment. (n = 3). (R) Agarose gel electrophoresis was performed to verify the qRT‐PCR products. (S) IGF1 mRNA expression in HT22 cells transfected with wild‐type and mutant Gm26550 overexpression plasmids. (n = 4). (T‐U) Representative immunoblots showing the effects of Gm26550 mutants. (i) Gm26550‐mut‐miR indicates that the miR‐26a‐5p binding site is mutated; (ii) Gm26550‐mut‐Δ359‐410 refers to deletion/truncation of the 359–410 region; and (iii) Gm26550‐mut‐(miR+Δ359‐410) contains both the mutated miRNA binding site and the truncated region. (n = 4). Data are presented as the mean ± SEM. *p < 0.05, **p < 0.01, ***p < 0.001, ^#^ *p* < 0.05, ^##^ *p* < 0.01, and ^###^ *p* < 0.001 by two‐tailed t test (A, F, and J) or two‐way ANOVA (D, G, Q, S, and U) or one‐way ANOVA (I, K, M, O, and P) followed by Bonferroni's post hoc test.

In addition to acting as competing endogenous RNAs (ceRNAs), lncRNAs can also regulate disease progression by interacting with RNA‐binding proteins (RBPs). Using multiple bioinformatics databases, including RBPmap, RBPDB, catRAPID, and RNA‐Protein Interaction Prediction (RPISeq), we predicted four candidate RBPs potentially interacting with Gm26550 (Figure S7A–C). Protein‐protein interaction (PPI) network analysis further identified KHSRP as the most prominent hub gene among these candidates (Figure S7D). To experimentally validate the interaction, RIP–qRT‐PCR assays were then performed in HT22 cells. Compared with the IgG control group, Gm26550 was significantly enriched in KHSRP immunoprecipitates (Figure S7E, F). Consistently, in the hippocampal tissues of WT mice, Gm26550 was also enriched in the KHSRP‐containing immunoprecipitates (Figure 6A and Figure S7G). RNA pull‐down assays further confirm the direct association between Gm26550 and KHSRP. In line with the RIP results, KHSRP protein was efficiently enriched by the Gm26550‐specific probe in hippocampal tissues from Nrf2^−/−^ mice (Figure 6B) and in HT22 cell lysates, supporting direct interaction under both in vitro and in vivo conditions (Figure S7H). Moreover, FISH combined with immunofluorescence staining showed that Gm26550 and KHSRP colocalized in the cytoplasm (Figure 6C). To determine whether Gm26550 regulates KHSRP expression, HT22 cells were subjected to Gm26550 overexpression or knockdown. Neither manipulation significantly affected KHSRP expression levels (Figure S7I–N).

KHSRP promotes AU‐ or U‐rich RNA element (ARE)‐directed cytoplasmic mRNA turnover by binding to AU‐ or U‐rich RNA elements (AREs) within the 3′untranslated region (3′UTR) of target transcripts and recruiting the degradation machinery [21]. Notably, IGF1 mRNA contains a long U‐ and AU‐rich 3′UTR harboring several predicted KHSRP binding motifs (Figure S8A–C). We therefore examined whether KHSRP regulates IGF1 mRNA stability and expression. First, the knockdown and overexpression efficiencies of KHSRP were confirmed in HT22 cells (Figure S8D,E). Actinomycin D chase assays showed that KHSRP knockdown prolonged the transcript half‐life of IGF1 compared to NC cells (Figure 6D). Consistently, qRT‐PCR revealed that KHSRP knockdown increased IGF1 mRNA levels (Figure S8F), and Western blot analyses demonstrated increased IGF1 protein expression relative to the NC group (Figure 6E,F). Conversely, KHSRP overexpression shortened the transcriptional half‐life of IGF1 and reduced both IGF1 mRNA and protein levels compared with the Vector group (Figure S8G–J). To assess whether Gm26550 regulates IGF1 via KHSRP, rescue experiments were performed. The reduction in IGF1 mRNA stability induced by Gm26550 knockdown was reversed by concomitant KHSRP knockdown (Figure 6G). Furthermore, KHSRP knockdown reversed the decreases in IGF1 mRNA and protein expression caused by Gm26550 knockdown in HT22 cells (Figure 6H,I and Figure S8K). To further validate the direct interaction between KHSRP and IGF1 mRNA, RIP assays with an anti‐KHSRP antibody were performed in hippocampal tissues of WT mice. Compared with the IgG control, IGF1 mRNA was significantly enriched in KHSRP‐containing immunoprecipitates (Figure 6J). Consistently, IGF1 mRNA was also enriched in KHSRP immunoprecipitates in HT22 cells (Figure S8L). Bioinformatics analysis using RBPmap identified a cluster of potential KHSRP binding sites within the 378–428 nt region of the IGF1 3′ UTR, with peak confidence scores at positions 403 and 428 (Z‐score > 2.5, *p* < 0.01; Figure S8M). To validate these potential interactions, we constructed luciferase reporter vectors containing either the wild‐type (WT) or a mutant (Mut) IGF1 3′ UTR, in which the identified critical ARE motifs were site‐specifically disrupted. Dual‐luciferase reporter assays revealed that KHSRP overexpression significantly suppressed the activity of the WT reporter, whereas this inhibitory effect was abrogated in the mutant construct (Figure 6K). Collectively, these findings indicate that the KHSRP‐mediated regulation of IGF1 relies on the integrity of these specific ARE motifs.

To identify the Gm26550 region responsible for binding to KHSRP, RNA pull‐down assays were performed in HT22 cells using three Gm26550 fragments (101–152, 251–302, and 359–410 nt) predicted by catRAPID prediction (Figure S9A–D). Among them, the 359–410 nt fragment exhibited the strongest binding to KHSRP (Figure 6L). Deletion of this fragment (Gm26550 Δ359‐410) markedly reduced binding to KHSRP compared with the full‐length transcript (Figure 6M and Figure S9E), indicating that the 359–410 nt region of Gm26550 is critical for KHSRP binding. We further mapped which KH domains of KHSRP are involved in Gm26550 binding using RIP assays in HEK293T cells expressing truncated KHSRP mutants lacking specific KH domains (Figure 6N). qRT‐PCR showed that deletion of either the KH3 or KH4 domain significantly decreased the amount of co‐immunoprecipitated Gm26550 (Figure 6O and Figure S9F). Consistently, when the KH3 or KH4 domain was deleted, the amount of co‐immunoprecipitated IGF1 mRNA was significantly reduced (Figure 6P and Figure S9G).

To further evaluate whether Gm26550 affects the association between KHSRP and IGF1 mRNA, RIP assays were performed using hippocampal tissues from Nrf2^−/−^ mice. The results showed that, compared with the WT group, the enrichment of Gm26550 in KHSRP immunoprecipitates was decreased in the hippocampal tissues of Nrf2^−/−^ mice, whereas IGF1 mRNA levels were increased (Figure 6Q,R). Consistently, in HT22 cells, Gm26550 knockdown reduced the endogenous amount of Gm26550 co‐precipitated with KHSRP and increased the enrichment of IGF1 mRNA in KHSRP immunoprecipitates (Figure S9H,I). These findings suggest that Gm26550 may function as a molecular decoy, thereby preventing KHSRP from negatively regulating IGF1 mRNA stability and expression.

To determine whether the miR‐26a‐5p‐ and KHSRP‐associated regions of Gm26550 both contribute to IGF1 regulation, HT22 cells were transfected with plasmids expressing WT Gm26550, Gm26550 carrying a mutant miR‐26a‐5p binding site, Gm26550 lacking the KHSRP‐binding region at 359–410 nt, or the corresponding double mutant. Compared with the empty‐vector control, WT Gm26550 overexpression significantly increased IGF1 expression (Figure 6S–U). In contrast, mutation of the miR‐26a‐5p binding site or deletion of the KHSRP‐binding region each partially attenuated the ability of Gm26550 to upregulate IGF1. Furthermore, simultaneous disruption of both regions produced a stronger reduction in IGF1 expression than either single mutation alone (Figure 6S–U).

Bioinformatic analysis identified the miR‐26a‐5p binding site at 1026–1051 nt, which is spatially distinct from the KHSRP‐associated region (Figure S10A,B). Interestingly, RNAfold predictions suggested an asymmetric structural response: while deletion of the KHSRP‐associated region did not appreciably affect the predicted conformation of the miR‐26a‐5p site, mutation of the miR‐26a‐5p site altered the predicted local structure around the KHSRP region (Figure S10C–E). Because such conformational coupling in vivo cannot be completely excluded, we conclude that both the miR‐26a‐5p site and the KHSRP‐binding region contribute to Gm26550‐mediated upregulation of IGF1.

### 2.6 Gm26550 Rescues Memory Impairments and Synaptic Plasticity in Nrf2^−/−^ Mice by Promoting IGF1 Expression

**FIGURE 7:**
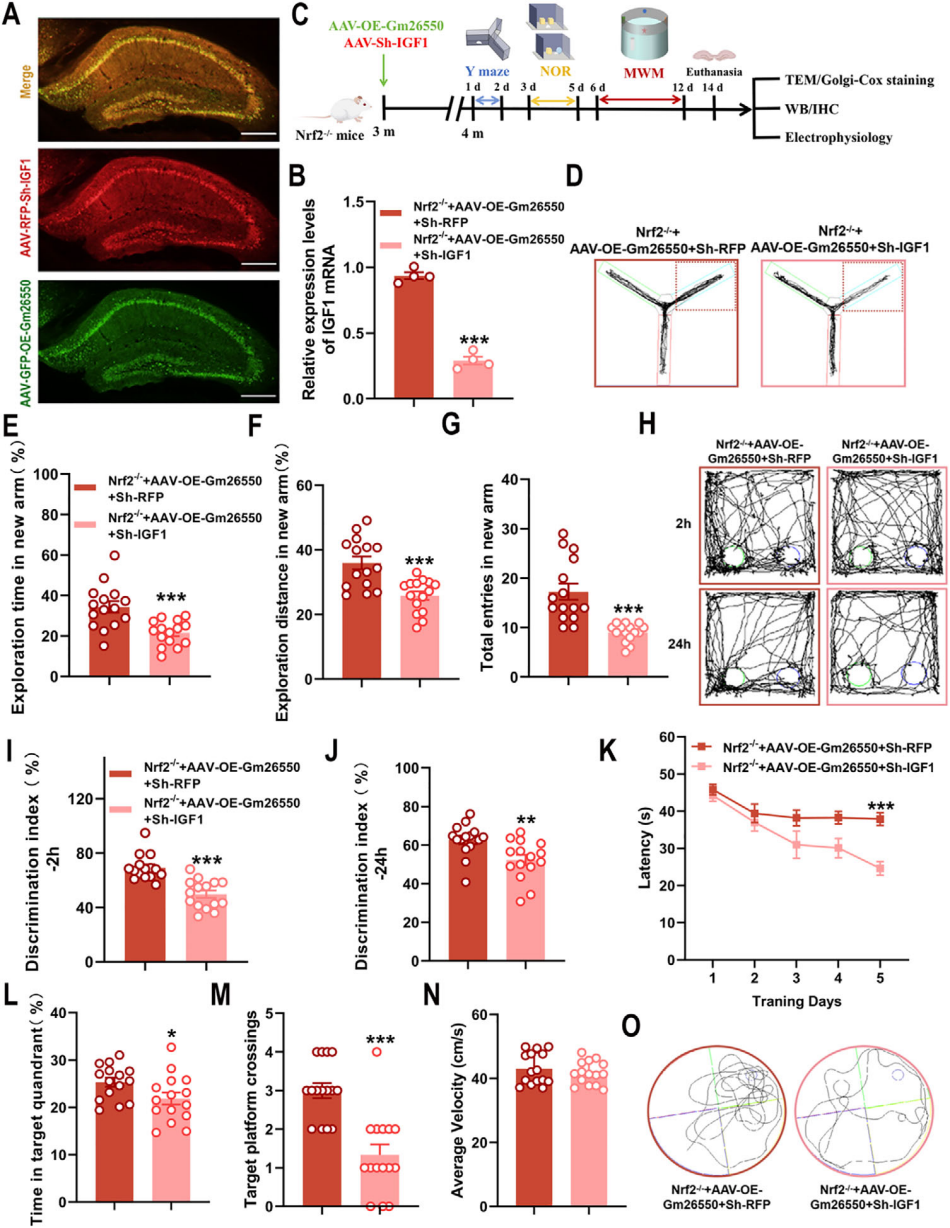
Gm26550 rescues memory impairments in Nrf2^−/−^ mice via IGF1. (A) Representative fluorescence images show GFP expression (indicating Gm26550 overexpression) and RFP expression (indicating IGF1 knockdown) in the hippocampus after stereotaxic injection. (Scale bars = 500 µm). (B) qRT‐PCR was used to detect the knockdown efficiency of IGF1 mRNA in the hippocampal region of Nrf2^−/−^ mice with Gm26550 overexpression. (n = 4). (C) The schematic diagram illustrates the experimental timeline. (D‐G) The Y‐maze test shows the percentage of time spent and distance traveled in the new arm. (n = 15). (H–J) NOR test was performed at 2 h and 24 h to evaluate the preference of the mice for familiar versus novel objects. (n = 15). (K–O) Behavioral performance of all mice in the MWM task. (n = 15). (K) Escape latency time (average latency) from Days 2 to 6. (L,M) Time spent in the target quadrant and the number of platform crossings on Day 7. (N) Average swimming velocity during the probe trial. (O) Representative swimming trajectories from two groups on Day 7. (n = 15). Data are presented as the mean ± SEM. *p < 0.05, **p < 0.01, and ***p < 0.001 by two‐tailed *t*‐test (B, E, F, G, I, J, L, M, and N) or two‐way ANOVA (K) followed by Bonferroni's post hoc test.

To explore whether the function of Gm26550 in learning and memory is mediated by IGF1, we knocked down IGF1 in the hippocampus of Nrf2^−/−^ mice that overexpressed Gm26550 (Figure 7A,B). Mice from these groups were then subjected to a series of behavioral tests, including the Y‐maze test, NOR, and the MWM test, to assess learning and memory performance (Figure 7C). In the Y‐maze test, IGF1 knockdown abolished the beneficial effects of Gm26550 overexpression on the time spent in the novel arm, the distance traveled in the novel arm, and the number of entries into the novel arm (Figure 7D–G). In the ORT, IGF1 knockdown also inhibited the increased DI induced by Gm26550 overexpression at 2 h and 24 h (Figures 7H–J). In the MWM, hippocampal IGF1 knockdown in Gm26550‐overexpressing Nrf2^−/−^ mice increased escape latency during the training phase (Figure 7K) and reduced the time spent in the target quadrant during the probe trial (Figure 7L). Moreover, IGF1 knockdown decreased the number of platform crossings (Figure 7M,O). In contrast, there was no significant effect on the average swimming velocity during the visible platform phase, indicating that the observed behavioral deficits were not due to motor impairment (Figure 7N).

**FIGURE 8:**
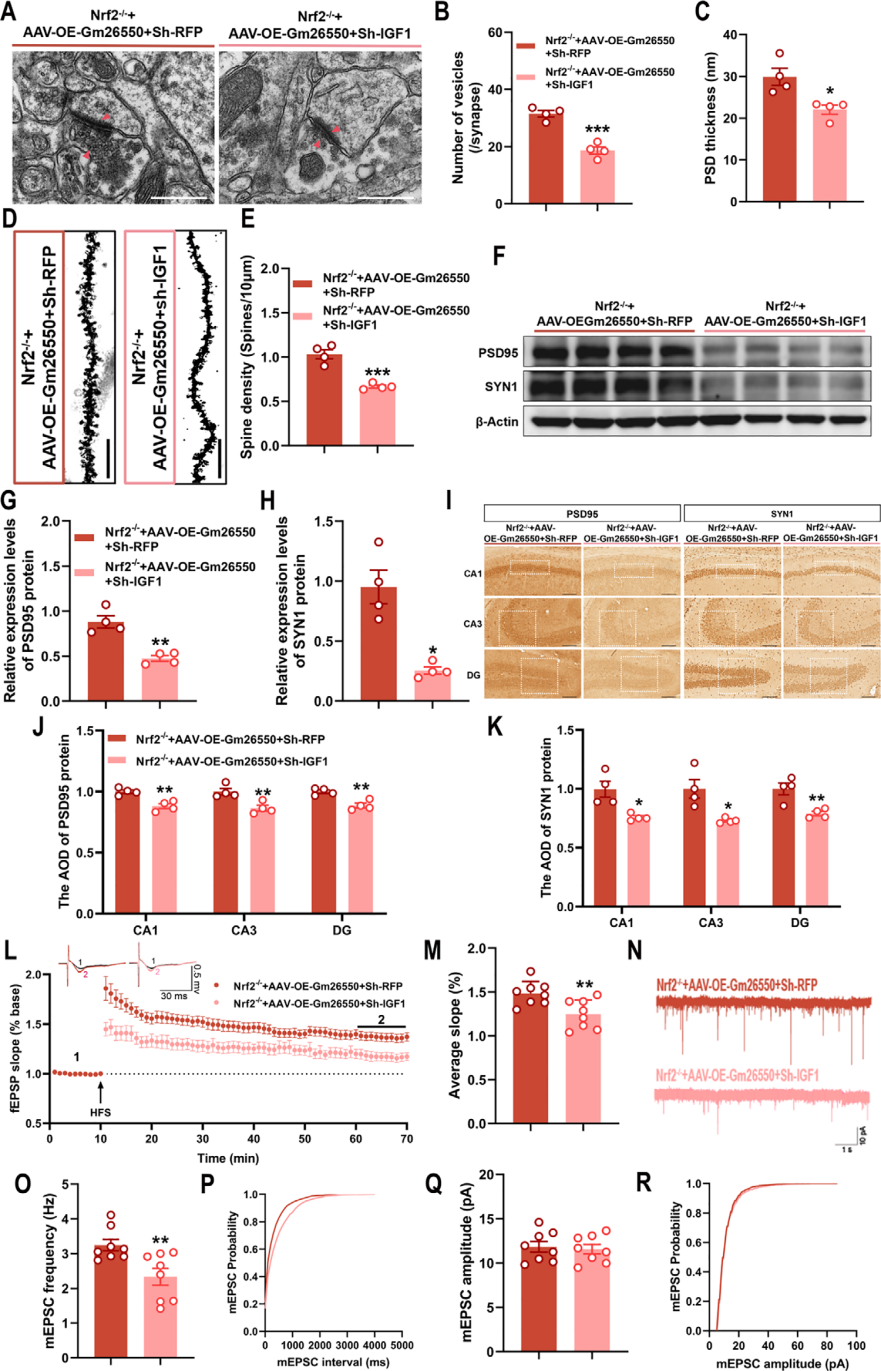
Gm26550 rescues synaptic plasticity in Nrf2^−/−^ mice via IGF1. (A) Representative TEM images of the hippocampal CA1 region in the two experimental groups of mice. (n = 4; Scale bars = 1 µm). (B,C) Quantification of TEM observations: the number of synaptic vesicles (B) and the thickness of the postsynaptic density (C). (D) Golgi‐Cox staining shows dendritic spines in the hippocampal CA1 region. (Scale bars = 10 µm). (E) Quantification of spine density. (n = 20 neurons from 4 mice). (F–H) Representative Western blot images showing PSD95 and SYN1 expression in the hippocampus. (n = 4). (I–K) Representative images and quantification of SYN1 and PSD95 IHC in the hippocampus of the two groups. (n  =  4, Scale bars  =  50 µm). (L) HFS‐induced LTP was recorded at the Schaffer collateral‐CA1 pathway in mice from the two experimental groups. The arrow indicates the time point of LTP induction. (M) Quantitative of the average fEPSP slope at 60 min. (n = 8 slices from 3 mice). (N–R) Representative traces of mEPSCs (N) and quantification of mEPSC frequency (O,P) and amplitude (Q,R) recorded from hippocampal CA1 pyramidal neurons. (n = 8 cells from 3 mice). Data are presented as the mean ± SEM. *p < 0.05, **p < 0.01, and ***p < 0.001 by two‐tailed *t*‐test (B, C, E, G, H, J, K, M, O, and Q).

Consistent with the behavioral findings, TEM analysis showed that IGF1 knockdown in the hippocampus of Nrf2^−/−^ mice overexpressing Gm26550 reduced the number of synaptic vesicles and decreased the thickness of the postsynaptic density in CA1 (Figure 8A–C). Golgi‐Cox staining further revealed that IGF1 knockdown reduced dendritic spine density in these mice (Figure 8D,E). Western blot analysis and IHC demonstrated that IGF1 knockdown significantly decreased the expression of PSD95 and SYN1 in the hippocampus of Nrf2^−/−^ mice overexpressing Gm26550 (Figure 8F–K). Functionally, IGF1 knockdown abolished the enhanced average fEPSP slope conferred by Gm26550 overexpression (Figure 8L,M), and it also eliminated the increased mEPSP frequency (Figure 8N–R). Collectively, these data indicate that the beneficial effects of Gm26550 on learning and memory, as well as synaptic plasticity, depend on IGF1.

## 3 Discussion

In the current study, we identified the role of the previously uncharacterized lncRNA Gm26550 in learning and memory impairments in Nrf2^−/−^ mice. Knockout of Nrf2 results in decreased Gm26550 expression in the hippocampus at the transcriptional level. Moreover, Gm26550 overexpression increases IGF1 expression through two mechanisms. First, Gm26550 functionally antagonizes miR‐26a‐5p‐mediated repression of IGF1. Second, Gm26550 binds the KH3/KH4 domains of KHSRP to block KHSRP‐dependent degradation of IGF1 mRNA. These two mechanisms jointly increase IGF1 expression, enhance synaptic plasticity in hippocampal neurons, and ultimately ameliorate learning and memory impairments.

This work revealed that Gm26550, transcriptionally activated by Nrf2, plays a pivotal role in regulating learning and memory as well as synaptic plasticity. As a key transcription factor, Nrf2 plays a vital role in cognitive impairments associated with aging and neurodegenerative diseases [22, 23, 24]. In the present study, we observed learning and memory impairments in Nrf2^−/−^ mice, which is consistent with previous findings [12]. Upon exposure to diverse adverse stimuli, Nrf2 binds to the ARE and promotes the transcription of neighboring target genes [8, 9]. As established, Nrf2 regulates synaptic plasticity and cognitive function through modulating oxidative stress, iron metabolism, as well as coordinating mitochondrial function [25, 26]. However, the specific mechanisms by which Nrf2 governs learning and memory remain unclear. Current therapeutic strategies aimed at targeting Nrf2 primarily rely on Nrf2 activators, including natural compounds such as curcumin and synthetic agents such as oltipraz. Nevertheless, these approaches have notable limitations. Curcumin has an oral bioavailability of less than 1% and poor water solubility, resulting in inadequate tissue penetration; oltipraz, in contrast, interferes with immune‐inflammatory pathways [27, 28]. Therefore, further investigation into the downstream effectors of the Nrf2 pathway is warranted.

Thus far, most studies on lncRNAs have shown that they participate in regulating neuronal function, synaptic plasticity, and learning and memory [17, 18, 19]. In mouse hippocampal neurons, lncRNA Gm12371 is upregulated by cyclic adenosine monophosphate (cAMP)‐protein kinase A (PKA) signaling and is essential for activity‐dependent changes in synaptic transmission [29]. Similarly, lncRNA NEAT1 mediates neuronal histone methylation and age‐related memory impairment [30]. In the present study, we found that Gm26550 was significantly downregulated in the hippocampus of Nrf2^−/−^ mice and was positively correlated with the DI, suggesting that Gm26550 may contribute to learning and memory processes. To elucidate its biological function, we used an AAV vector to overexpress Gm26550 in the hippocampus of Nrf2^−/−^ mice. We then conducted a series of behavioral tests. The results showed that Gm26550 overexpression ameliorated learning and memory impairments in Nrf2^−/−^ mice. Synaptic plasticity is considered the foundation of learning and memory, including both structural and functional changes at synapses [31]. Notably, by observing synaptic ultrastructure via electron microscopy, performing Golgi staining, assessing the expression of postsynaptic proteins, and conducting electrophysiological recordings, we confirmed that Gm26550 overexpression enhances synaptic plasticity in Nrf2^−/−^ mice, thereby supporting its role in regulating neuronal structure and function.

To further explore how Gm26550 may contribute to synaptic plasticity‐related phenotypes, we intersected hippocampal mRNAs differentially expressed in Nrf2^−/−^ mice with genes annotated in the SynGO database and identified 30 synapse‐associated candidates. Correlation analysis between Gm26550 and these candidates highlighted IGF1 as a potentially important downstream effector. This observation provided a rationale for focusing on the Gm26550–IGF1 regulatory axis, although correlation analysis alone cannot establish causality.

IGF1, a key member of the insulin superfamily, is an important regulator of learning, memory, and synaptic plasticity, and its expression is closely associated with cognitive function [32, 33]. In the hippocampus, IGF1 is mainly synthesized and secreted by pyramidal neurons in the CA1 region [32]. Activity‐dependent release of IGF1 enables it to bind to IGF1 receptors on the same neuron, thereby activating downstream signaling pathways, including the phosphatidylinositol 3‐kinase/protein kinase B pathway and the mitogen‐activated protein kinase/extracellular signal‐regulated kinase pathway [34]. These signaling cascades promote dendritic spine remodeling and contribute to the induction and maintenance of long‐term potentiation by regulating synaptic plasticity‐related genes, modulating the abundance and activity of neurotransmitter receptors at the postsynaptic membrane, and potentially influencing presynaptic neurotransmitter release [35]. Conversely, inhibition of IGF1 synthesis or autocrine IGF1 signaling in hippocampal CA1 pyramidal neurons impairs synaptic plasticity [35]. Consistent with these findings, reduced IGF1 levels have been associated with cognitive impairment, whereas higher IGF1 levels are generally linked to better cognitive performance [36].

In the present study, we observed a significant reduction in IGF1 expression in the hippocampus of Nrf2^−/−^ mice, consistent with the involvement of IGF1 deficiency in synaptic dysfunction. Importantly, hippocampal overexpression of Gm26550 significantly increased IGF1 expression, and consistent results were obtained in cellular experiments. These findings support the possibility that Gm26550 contributes to synaptic plasticity regulation, at least in part, through modulation of IGF1.

Furthermore, we sought to elucidate the mechanism by which Gm26550 regulates IGF1. Previous studies have demonstrated that lncRNAs regulate gene expression through diverse molecular modes, including DNA modification, RNA‐RNA interactions, and protein binding [37]. Notably, the potential mechanisms of lncRNA action can be inferred from their subcellular localization [38]. For example, the nuclear‐localized lncRNA ADRAM regulates fear extinction through direct interaction with the chaperone protein 14‐3‐3 [39]. In a mouse model of depression, the cytoplasm‐localized lncRNA Gm2694 is highly expressed in the hippocampus [40]. By binding to the endoplasmic reticulum (ER) stress protein GRP78, Gm2694 disrupts ER homeostasis, thereby inhibiting presynaptic glutamate release [40]. Knockdown of Gm2694 restores synaptic transmission efficiency and LTP, while alleviating depressive‐like behaviors and spatial learning and memory deficits [40]. In our study, FISH revealed that Gm26550 is predominantly localized in the cytoplasm, suggesting that it exerts its functions in this compartment. We therefore propose that Gm26550 sustains IGF1 abundance through two distinct cytoplasmic regulatory modes: functional antagonism of miRNA and physical sequestration of RNA‐binding protein. Through these dual pathways, Gm26550 ultimately rescues both synaptic plasticity defects and learning‐memory impairments in Nrf2^−/−^ mice. Through these dual pathways, Gm26550 ultimately rescues both synaptic plasticity defects and learning‐memory impairments in Nrf2^−/−^ mice. Nevertheless, the weak nuclear signal observed in FISH assays suggests that Gm26550 may also be involved in additional, yet uncharacterized nuclear functions. It is therefore possible that Gm26550 could contribute to transcriptional regulation through chromatin association, which may add an additional layer of complexity to the Nrf2–Gm26550 regulatory network. Future studies employing chromatin mapping approaches such as ChIRP‐seq could determine whether Gm26550 directly associates with chromatin and regulates transcription of IGF1 or other downstream targets.

In the maintenance of neuronal morphology and synaptic plasticity, miRNAs have been identified as common targets of circRNAs or lncRNAs, and several of these miRNAs play critical roles in synaptic function [41, 42]. Here, we first performed cross‐screening using multiple bioinformatic databases to identify miRNAs predicted to target both Gm26550 and IGF1. miR‐26a‐5p was of particular interest because it is downregulated in the prefrontal cortex and hippocampus and has been closely implicated in memory formation [43]. Although the expression profile of miR‐26a‐5p in the hippocampus under learning‐ and memory‐related disorders remains unclear, several studies have reported that miR‐26a‐5p is upregulated in the hippocampus of Nrf2^−/−^ mice [12], as well as in the blood samples from AD patients [44]. This suggests that miR‐26a‐5p may serve as a potential biomarker and be associated with learning and memory impairments. Our data demonstrated that the upregulation of miR‐26a‐5p in Nrf2^−/−^ mice depends on Gm26550. Notably, the upregulation of miR‐26a‐5p observed in Nrf2^−/−^ mice was rescued by overexpression of Gm26550. Consistently, in vitro cellular assays further confirmed these regulatory relationships. In other studies, miR‐26a‐5p may be regulated by distinct transcription factors [44], inflammatory signals [45], or stress‐responsive pathways [46], leading to context‐dependent biological outcomes. Our study identified miR‐26a‐5p as a downstream target of Gm26550, which is directly regulated by Nrf2. This provides a distinct upstream regulatory axis in our experimental system, with Nrf2 serving as the key upstream transcription factor governing the Gm26550‐miR‐26a‐5p cascade, which is distinct from upstream regulators reported previously. Nevertheless, ceRNA efficacy in vivo is influenced by the relative abundance of RNAs and subcellular compartmentalization. In the present study, the absolute copy numbers (stoichiometry) of Gm26550, miR‐26a‐5p, and IGF1 in hippocampal neurons were not quantified. Therefore, whether this sponge‐like interaction reaches quantitative physiological sufficiency remains to be determined in future studies using absolute quantification approaches (e.g., ddPCR or digital RT‐qPCR).

It is well established that miRNAs exert pleiotropic post‐transcriptional regulatory functions, as a single miRNA can simultaneously target numerous distinct mRNA transcripts. Consequently, the biological output of any given miRNA is highly context‐dependent, varying across cell types, tissues, and physiological or pathological states. Prior work has validated miR‐26a‐5p as a suppressor of Phosphatase and tensin homolog (PTEN); relief of PTEN‐mediated inhibition subsequently activates the PI3K/Akt cascade to enhance synaptic plasticity, learning, and memory [42]. In the current study, we combine bioinformatic prediction and biochemical validations to identify IGF1 as another target of miR‐26a‐5p. In vitro functional assays further confirm that miR‐26a‐5p robustly represses IGF1 expression, and we demonstrate that the miR‐26a‐5p/IGF1 signaling axis impairs neuronal plasticity and cognitive performance—an opposing, anti‐plasticity phenotype relative to the pro‐cognitive effects described in earlier PTEN‐focused reports. Notably, PTEN and IGF1 transcripts coexist within the same neuronal populations, meaning miR‐26a‐5p can simultaneously engage both target mRNAs in a single cell. We therefore propose that the net physiological effect of miR‐26a‐5p on synaptic plasticity is dictated by three interrelated factors: (1) the relative cellular abundance of PTEN and IGF1 transcripts; (2) the differential structural accessibility of miR‐26a‐5p binding motifs on each target mRNA; and (3) the functional dominance of the downstream PTEN/PI3K‐Akt versus IGF1 signaling axes under distinct physiological or pathological conditions. Collectively, these data illustrate that miR‐26a‐5p elicits divergent, even opposing, biological outcomes by repressing distinct downstream effector genes, with its final phenotype shaped by target‐specific context within neurons.

Previous studies have revealed that lncRNAs frequently modulate downstream targets by interacting with RBPs, which serve as critical regulators of transcription and translation [47, 48]. Several lncRNAs, including NEAT1 [49], H19 [50], EPR [51], AB074169 [52], and ALAE [53], have been reported to interact with KHSRP and regulate mRNA stability, translation, or splicing. By combining bioinformatic analyses and experimental validation, we demonstrated that KHSRP is a key interacting partner of Gm26550 in HT22 cells. Notably, neither Nrf2 deficiency nor Gm26550 overexpression or knockdown altered KHSRP protein expression in the hippocampus or HT22 cells, suggesting that Gm26550 modulates KHSRP activity through direct physical interaction rather than by changing KHSRP expression levels. In the present study, we found that Gm26550 binds to the KH3/KH4 domains of KHSRP via its 359–410 nt. The lincRNA H19 has been shown to directly bind the KH1 domain of KHSRP and facilitate degradation of Myogenin mRNA during myogenic differentiation [51], highlighting distinct KHSRP‐related functions mediated by different lncRNAs through specific RNA‐binding domains.

KHSRP is a multifunctional RBP that negatively regulates neuronal development and memory consolidation by binding to and promoting the degradation of mRNAs associated with axon growth and synaptic function, including GAP43 and SNAP25 [54, 55]. KHSRP specifically recognizes and binds to AREs within the 3′UTR of target mRNAs, thereby recruiting these transcripts to the cytoplasmic RNA exosome complex and facilitating ARE‐dependent mRNA decay [21, 56, 57]. Notably, the 3′UTR of IGF1 mRNA is enriched in ARE sequences. Moreover, we observed that KHSRP regulates IGF1 mRNA stability via its KH3/KH4 domains in HT22 cells. We therefore suggest that KHSRP may modulate IGF1 mRNA stability and expression through an ARE‐dependent degradation pathway. While our rescue experiments establish IGF1 as a key mediator of the Gm26550‐KHSRP axis, it remains possible that additional KHSRP‐regulated transcripts, such as those encoding synaptic proteins, also contribute to the observed phenotypes. Unbiased transcriptomic screening following KHSRP manipulation in hippocampal neurons would help delineate the full repertoire of KHSRP‐regulated mRNAs in this context. Interestingly, we found that Gm26550 and IGF1 mRNA interact with the same RNA‐binding domain of KHSRP, suggesting that Gm26550 may interfere with KHSRP binding to IGF1 mRNA. We further propose that Gm26550, through preferential binding to the KH3/KH4 domains, could displace KHSRP and thereby reduce KHSRP‐mediated exosome targeting of IGF1 mRNA.

A key mechanistic question raised by our findings is the spatial and functional relationship between the miR‐26a‐5p binding sites and the KHSRP‐interacting region on Gm26550. Our detailed mapping reveals that these regulatory elements are located at distinct positions: the miR‐26a‐5p binding site (1026–1051 nt) does not overlap with the KHSRP‐binding region (359–410 nt), suggesting the potential for simultaneous engagement of both miR‐26a‐5p and KHSRP by a single Gm26550 molecule. To functionally test this, we employed a series of Gm26550 mutants. Disruption of either the miR‐26a‐5p binding site or the KHSRP‐binding region individually resulted in a partial loss of Gm26550's ability to enhance IGF1 expression, while concurrent disruption of both sites in the double mutant led to a greater reduction in activity. Our observation of no significant interaction between the two mutations at either the mRNA or protein level strongly supports an additive model, indicating that these mutations may act independently on the examined scale. However, given the moderate sample size, this analysis is underpowered to definitively establish additivity. Moreover, the Δ359‐410 deletion may influence local RNA conformation and potentially affect distal miR‐26a‐5p site accessibility, as suggested by RNAfold predictions. Therefore, while our data support the functional contribution of both modules, we refrain from concluding that they operate through strictly independent pathways. These separation‐of‐function mutant experiments were performed under overexpression conditions and do not fully establish endogenous‐level independence of the miR‐26a‐5p‐ and KHSRP‐dependent pathways. Future studies employing endogenous motif‐specific editing and higher‐resolution structural probing (e.g., SHAPE‐MaP) will be necessary to resolve their precise spatial and functional relationship under physiological conditions.

One limitation of this study is the exclusive use of male mice, necessitating future sex‐stratified validation. Furthermore, while our separation‐of‐function mutants suggest the existence of two distinct regulatory modules, we did not orthogonally verify that each single mutant selectively ablates only its cognate interaction (e.g., whether the miR‐site mutant retains KHSRP binding or if the KHSRP‐deletion mutant preserves AGO2 association), which precludes a definitive conclusion regarding their mechanistic independence. At the cellular level, although our core findings were corroborated in vivo in Nrf2‐deficient hippocampal tissue, the mechanistic assays—namely the RIP analysis—were not replicated in primary hippocampal neurons, leaving the cell‐autonomous nature of this regulation partially unaddressed. Moreover, our phenotypic characterization was not complemented by standalone IGF1 sufficiency assays to establish a direct causal link. Collectively, future investigations incorporating IGF1 supplementation, targeted Gm26550 manipulation, and orthogonal mutant validation will be essential to fully establish the broader biological significance and therapeutic potential of the Gm26550/IGF1 axis.

In summary, our study establishes the cytoplasmic lncRNA Gm26550 as a critical regulator of hippocampal synaptic plasticity and cognitive function, acting through dual post‐transcriptional mechanisms—miR‐26a‐5p antagonism and KHSRP sequestration—to sustain IGF1 expression and mRNA stability. While our findings provide a mechanistic framework, they also suggest that Gm26550 warrants further investigation as a potential biomarker for cognitive dysfunction. Moreover, the functional interplay identified here offers a conceptual basis for developing therapeutic strategies targeting the Gm26550/IGF1 axis to ameliorate learning and memory deficits in conditions such as Nrf2 deficiency.

## 4 Experimental Section

### 4.1 Human Samples

Human Samples Human brain tissue samples were obtained from the Human Brain Bank of Hebei Medical University, Shijiazhuang, China. All donors or their legal representatives provided written informed consent for the use of brain tissue and associated clinical data for scientific research. This study was approved by the Medical Ethics Committee of Hebei Medical University (Approval No. 2025012). The criteria for AD diagnosis were based on previous studies [58], and the operational protocols were conducted as previously described [59]. Detailed information is presented in Table S13.

### 4.2 Animals

Adult male wild‐type (WT) mice with an ICR background (8–12 weeks old) were purchased from SPF Biotechnology Co., Ltd. (Beijing, China). Nrf2 knockout (Nrf2^−/−^) mice with an ICR background were kindly provided by Professor Chunyan Li (Department of Neurology, the Second Hospital of Hebei Medical University, Shijiazhuang City, Hebei Province, China). Male wild‐type (WT) mice (C57BL/6J background, 4–8 weeks old) were purchased from SPF Biotechnology Co., Ltd. (Beijing, China), and 5×FAD mice (C57BL/6J background) were obtained from The Jackson Laboratory (Bar Harbor, ME, USA). Genotypes of Nrf2^−/−^ and WT mice were determined by PCR amplification of genomic DNA isolated from tail tissue, as described in our previous study [10]. All animals were housed under specific pathogen‐free (SPF) conditions with a 12 h light/dark cycle and had ad libitum access to food and water. Mice were acclimated to the facility for 1 week prior to experimentation. All animal procedures were performed in compliance with the Animal Welfare Act and were approved by the Medical Ethics Committee of Hebei Medical University (IACUC‐Hebmu‐P‐2024165). Efforts were made to minimize animal suffering and reduce the number of animals used. Animals with pre‐existing health impairments were excluded from the study. To minimize experimental bias, a rigorous double‐blind protocol was implemented. In brief, the experimenters who performed the behavioral tests and data analyses were blinded to the group allocation. An independent researcher assigned the mice to groups and handled the animal coding. Unblinding was conducted only after the entire dataset was collected and the statistical analyses were finalized.

### 4.3 Y‐Maze Test

The Y‐maze apparatus consisted of three arms (30 cm × 8 cm × 15 cm) intersecting at a 120° angle. In each trial, the three arms were randomly designated as the unfamiliar arm, the start arm, and the reference arm. During the training phase, the arm designated as the unfamiliar arm for the subsequent test was closed. During the testing phase, the unfamiliar arm was opened to allow the mice to enter. The start arm was defined as the arm in which the mouse was placed at the beginning of the experiment. Throughout the testing phase, all three arms (start, unfamiliar, and reference) were kept open for 5 min, during which the animals were allowed to explore freely. Exploratory preference for the unfamiliar arm was calculated as the percentage of entries into the unfamiliar arm relative to the total number of entries into all three arms.

### 4.4 Novel Object Recognition (NOR) Test

To acclimate the mice to the test environment, they were placed in the behavioral testing room 24 h prior to the experiment. The NOR consisted of two phases: a training phase and a testing phase. During the training phase, two identical objects were placed at the left and right ends of one side wall in the testing arena. Each mouse was placed in the arena with its back facing the two objects and allowed to explore freely for 10 min. Twenty‐four hours later, one of the familiar objects was replaced with a novel object. The mouse was then allowed to explore the two distinct objects freely for 5 min. Finally, the discrimination index (DI) for the novel object was calculated as follows: DI = TN/(TF + TN), where TN is the time spent exploring the novel object and TF is the time spent exploring the familiar object.

### 4.5 Morris Water Maze (MWM) Task

Distinct visual cues were attached around the swimming pool. The water temperature was maintained between 20°C–22°C. Prior to formal training, each mouse underwent a pre‐test without the platform to exclude animals that were unable to adapt to the pool environment. Mice were trained in the water maze to find a hidden platform for 5 consecutive days, with four trials per day and a maximum trial duration of 60 s. If a mouse located the platform, it was allowed to remain on it for 15 s. If not, the mouse was guided to the platform and held there for 15 s. Movements were recorded using a video camera installed 1.5 m above the water surface on the room ceiling. On day 7, after 5 days of training, the hidden platform was removed, and a probe test was conducted. Each mouse was placed in the pool from the quadrant opposite the target quadrant and allowed to search for the former platform location for 60 s. The recorded parameters included the average escape latency (time to locate the platform), the percentage of time spent in the target quadrant, and the number of crossings over the former platform position.

### 4.6 Stereotactic Injection

Adeno‐associated viral (AAV) vectors, including the hippocampal‐targeted Gm26550 overexpressing vector (AAV‐OE‐Gm26550), the IGF1 knockdown vector (AAV‐Sh‐IGF1), and the corresponding control vectors (AAV‐GFP for overexpression control and AAV‐Sh‐RFP for the knockdown control), were constructed and supplied by Hanbio Biotechnology (Shanghai, China). The titer of all AAV vectors was 1.0 × 10^1^ ^3^ vector genomes (vg)/µL. Mice were anesthetized with isoflurane and fixed on a high‐precision stereotactic instrument (model 78–1311, KD Scientific, Holliston, MA, USA). Injection coordinates for the hippocampal CA1 region were determined using the mouse brain atlas (Paxinos & Franklin, 2001) as follows: anteroposterior (AP), −1.89 mm relative to bregma; mediolateral (ML), ±2.29 mm; dorsoventral (DV), −1.65 mm (measured from the skull surface). For single‐vector injections, 0.5 µL of AAV‐OE‐Gm26550 (experimental group) or 0.5 µL of AAV‐GFP (control group) was injected into the hippocampal CA1 region. For combinatorial vector injections, AAV‐OE‐Gm26550 was mixed with AAV‐Sh‐IGF1 at equal volumes to generate the AAV‐OE‐Gm26550 + Sh‐IGF1 group, and AAV‐OE‐Gm26550 was mixed with AAV‐Sh‐RFP (non‐targeting shRNA control) at equal volumes to generate the AAV‐OE‐Gm26550 + Sh‐RFP group. In both combinatorial groups, 1 µL of each mixed vector solution was injected into the hippocampus. All injections were delivered at a constant rate of 0.1 µL/min using a microsyringe. After injection, the needle was left in place for 15 min to minimize viral reflux, and then was slowly withdrawn. After surgery, mice were placed on a 37°C heated blanket until they regained consciousness and then returned to their home cages for post‐operative care. Behavioral and molecular experiments were performed 28 days after injection to allow stable AAV‐mediated transgene expression.

### 4.7 Transmission Electron Microscopy (TEM) Analysis

Experimental mice were anesthetized and transcardially perfused with pre‐cooled 0.9% normal saline to flush out the blood, followed by immediate perfusion with a pre‐cooled fixation solution containing 4% paraformaldehyde (PFA) and 2.5% glutaraldehyde. Hippocampal tissues were rapidly dissected immediately after perfusion, trimmed into 1 mm^3^ cubes, and immersion‐fixed in 4% PFA for 48 h at 4°C. Subsequent TEM sample preparation was conducted at the Transmission Electron Microscopy Laboratory, Large Instrument Experiment Platform of Hebei Medical University. Synaptic ultrastructure in the hippocampal region was examined and imaged using an HT7800 transmission electron microscope (Hitachi, Tokyo, Japan) operated at 80 kV. Synaptic structures were identified based on characteristic morphological features, including presynaptic terminals containing synaptic vesicles and postsynaptic densities (PSDs).

### 4.8 Golgi‐Cox Staining

Golgi‐Cox staining was performed using the Golgi Rapid Staining Kit (Cat#: GMS80020.1, GenMed Scientifics, USA) strictly according to the manufacturer's instructions. Hippocampal sections were fixed in the kit‐provided fixative for 14 days. Subsequently, 100‐µm‐thick sections were cut at room temperature using a Leica VT100S vibratome (Leica Biosystems, Wetzlar, Germany). The sections were processed through standard histological steps: graded ethanol dehydration (50%, 70%, 90%, 100%), xylene clearing, and mounting with a neutral mounting medium. Xylene was used for clearing prior to mounting. Stained sections were visualized using a Leica LED bright‐field microscope (Cat#: DM2000, Leica Biosystems, Wetzlar, Germany). For dendritic spine density quantification, pyramidal neurons in the hippocampal CA1 region were selected based on clear dendritic arborization and intact spine morphology. For each neuron, at least five non‐overlapping 10‐µm segments of apical secondary and tertiary dendrites were randomly selected for analysis. Spine density was calculated as the number of dendritic spines per 10 µm of dendritic length. All spine counting was performed independently by two blinded researchers using FIJI (ImageJ) software (National Institutes of Health, Bethesda, MD, USA). Inter‐observer consistency was assessed by cross‐validation of the counting results, with the coefficient of variation maintained below 10% to ensure reliable quantification.

### 4.9 Bioinformatics Analysis

#### 4.9.1 Microarray Data Processing

Published microarray transcriptome datasets of hippocampal tissues from wild‑type (WT) and Nrf2 knockout (Nrf2^−/−^) mice were retrieved for differential expression analysis of lncRNAs and mRNAs [10]. Transcripts with fold change (FC) > 2 and p < 0.05 were defined as differentially expressed lncRNAs (DElncRNAs), whereas those with FC > 1.5 and p < 0.05 were classified as differentially expressed mRNAs (DEmRNAs). Two forms of redundant entries were identified: identical lncRNA records and separate mRNA probes targeting distinct isoforms of the same gene but with divergent FC and P‑values. All redundant transcripts were highlighted in yellow in Tables S1,S2 and S5,S6. During deduplication, we retained one representative entry per gene: for lncRNAs, we kept one copy of identical duplicates; for mRNAs, we selected the probe with the smallest P‑value and the largest absolute FC. This process yielded 226 unique DElncRNAs (downregulated) and 401 unique DEmRNAs (both up‐ and downregulated) for downstream analysis (Tables S4, S7, and S8).

#### 4.9.2 Synaptic Gene Annotation and Ortholog Mapping

To prioritize synaptically relevant candidates, the identified mouse DEGs were first mapped to their human orthologs using the HomoloGene database and the biomaRt R package. The resulting human orthologs were then intersected with the SynGO database (https://syngoportal.org/), a curated resource of synaptic genes [60]. Genes with SynGO annotations were designated as high‑confidence synaptic candidates (Tables S9‐S10).

#### 4.9.3 Co‑Expression Network Analysis

To identify potential downstream effectors of Gm26550, we performed a co‑expression analysis using the normalized expression data from hippocampal samples (n = 6). Pearson correlation coefficients were calculated between Gm26550 and all annotated protein‑coding genes. Given the exploratory nature of this analysis with a limited sample size, we applied a liberal screening threshold of |r| ≥ 0.8 and *p* < 0.05 to identify genes with strong coordinated expression patterns. To further evaluate the robustness of these correlations, a nonparametric percentile bootstrap method with 2,000 resampling iterations was used to estimate the 95% confidence intervals (CIs) of the correlation coefficients. A correlation whose 95% CI did not cross zero was considered a stable co‑expression trend (Tables S11–S12).

### 4.10 Immunohistochemical (IHC) Staining

Experimental mice were anesthetized and transcardially perfused with pre‐cooled 0.9% normal saline, followed by pre‐cooled 4% PFA. Brain tissues were promptly dissected after perfusion, post‐fixed in 4% PFA at 4°C for 48 h, and subsequently embedded in paraffin. Paraffin‐embedded hippocampal tissues were sectioned into 5‐µm‐thick sections. Prior to staining, sections were dewaxed and rehydrated through a graded ethanol series (100%, 95%, 80%, and 70%) and then rinsed in distilled water. Antigen retrieval was performed by incubating sections in citrate buffer for 20 min, followed by natural cooling to room temperature (RT). All subsequent staining procedures were performed strictly according to the manufacturer's protocol for the Hypersensitive SP IHC Kit (Cat#: KIT‐9720, MXB Biotech, Fuzhou, China) to ensure standardized results. Briefly, endogenous peroxidase activity was blocked by adding a peroxidase blocker dropwise, followed by incubation for 10 min at room temperature. After three washes with phosphate‐buffered saline (PBS, pH 7.4) for 5 min each, non‐specific binding sites were blocked by incubating sections with a non‐specific binding blocker for 10 min at RT. Sections were then incubated overnight at 4°C with primary antibodies. The next day, sections were equilibrated to RT for 1 h and washed three times with PBS (5 min each). Subsequently, sections were incubated with a biotin‐labeled IgG polymer for 10 min at RT. After incubation with a streptavidin‐peroxidase conjugate for 10 min at RT, sections were washed three times with PBS (5 min each). Immunoreactivity was visualized by incubating sections with 3, 3’ ‐diaminobenzidine (DAB) chromogenic solution (Cat#: ZLI‐9018, Zhongshan Jinqiao Biotechnology, Beijing, China) for 2 min at RT. The reaction was stopped by immersing sections in distilled water. Sections were then dehydrated through a graded ethanol series (70%, 80%, 95%, and 100%), cleared in xylene, and mounted with a neutral mounting medium. Stained sections were observed using an Olympus BX43 bright‐field microscope (Olympus Corporation, Tokyo, Japan), and images were captured at consistent magnification across all samples. Quantitative analysis of immunoreactivity was performed using Image J software (National Institutes of Health, Bethesda, MD, USA). Average optical density (AOD) values were calculated as the quantitative indicator of protein expression levels. The antibodies used in this study are listed in Table S14.

### 4.11 Hippocampal Electrophysiological Recordings

Mice were anesthetized with isoflurane and then decapitated. Brains were rapidly excised and immersed in pre‐cooled cutting artificial cerebrospinal fluid (ACSF) continuously bubbled with 95% O_2_ and 5% CO_2_. The cutting ACSF contained (in mM): 93 N‐methyl‐D‐glucamine, 2.5 KCl, 1.2 NaH_2_PO_4_, 20 HEPES, 25 D‐glucose, 30 NaHCO_3_, 10 MgSO_4_, 0.5 CaCl_2_, 5 sodium ascorbate, 3 sodium pyruvate, and 2 thiourea (pH 7.3–7.4; osmolarity: ∼300 mOsm). Coronal hippocampal slices (300 µm thick) were prepared using a VT1200S vibratome (Leica Biosystems, Germany) and immediately transferred to an incubation ACSF containing (in mM): 93 NaCl, 2.5 KCl, 1.2 NaH_2_PO_4_, 20 HEPES, 25 D‐glucose, 30 NaHCO_3_, 2 MgSO_4_, 2 CaCl_2_, 5 sodium ascorbate, 3 sodium pyruvate, and 2 thiourea (pH: 7.3–7.4; osmolarity: ∼300 mOsm). Slices were incubated for at least 1 h at room temperature (24°C–26°C) and then transferred to a BX51WI fluorescence microscope (Olympus, Tokyo, Japan) for recordings. All recordings were performed in recording ACSF (in mM): 125 NaCl, 2.5 KCl, 1.2 NaH_2_PO_4_, 5 HEPES, 10 D‐glucose, 25 NaHCO_3_, 2 MgSO_4_, and 2 CaCl_2_ (pH: 7.3–7.4; osmolarity: ∼300 mOsm). During recordings, slices were continuously perfused with recording ACSF at a flow rate of 2–4 mL/min and maintained at 33 ± 0.5°C. Throughout the experiment, all ACSF solutions (cutting, incubation, and recording) were continuously bubbled with 95% O_2_ and 5% CO_2_ to maintain cell viability.

#### 4.11.1 Long‐Term Potentiation (LTP) Recordings

For LTP recordings, borosilicate glass recording electrodes (Sutter Instrument, Novato, CA, USA) were filled with recording ACSF filtered through a 0.22‐µm pore‐size filter. The stimulating electrode was placed on the Schaffer collateral pathway, and the recording electrode was positioned in the hippocampal CA1 region. The resistance of recording electrodes was 2–4 MΩ, and there were no air bubbles in the electrode lumen. The recording electrode was connected to an Axon Clamp amplifier (Molecular Devices, San Jose, CA, USA) via an electrode holder. Field excitatory postsynaptic potentials (fEPSPs) were converted from analog to digital signals using a Digidata 1440A system (Molecular Devices). Prior to baseline recording, an input‐output (I/O) curve was generated by gradually increasing the stimulus intensity from 0.02 to 0.1 mA to assess slice responsiveness and determine the optimal baseline stimulus intensity (set to evoke ∼50% of the maximum fEPSP amplitude). Baseline fEPSPs were recorded for 10 min at a stimulation frequency of 0.33 Hz. LTP was induced by theta burst stimulation (TBS), consisting of five trains of 100 Hz tetanic pulses (Each train containing four pulses at 100 Hz, with an inter‐train interval of 200 ms). After TBS induction, fEPSPs were continuously recorded for an additional 60 min using the same baseline stimulation parameters. LTP was considered successfully induced when the average fEPSP slope during the last 10 min of recording increased by more than 20% relative to the baseline period.

#### 4.11.2 Miniature Excitatory Postsynaptic Current (mEPSC) Recordings

For mEPSC recordings, glass recording electrodes were filled with internal solution containing (in mM): 135 CsMeSO_3_, 10 CsCl, 10 HEPES, 0.5 EGTA, 2 QX‐314, 4 Mg‐ATP, 0.4 Na_2_‐GTP, and 10 Na_2_‐Phosphocreatine (pH: 7.3–7.4; osmolarity: ∼300 mOsm). Recordings were performed in voltage‐clamp mode at a holding potential of −70 mV. To isolate mEPSCs, 1 µM tetrodotoxin (TTX) was added to the recording ACSF to block action potentials. Each recording lasted 3–5 min to ensure sufficient data acquisition. Acquired mEPSC events were analyzed using MiniAnalysis software (Synaptosoft, Decatur, GA, USA). The frequency (number of events per unit time) and amplitude of mEPSCs were quantified for statistical analysis.

### 4.12 Cell Culture

The mouse hippocampal neuronal cell line HT22 (Cat#: M8‐0201, Saiye Biological Technology, Suzhou, China, RRID: CVCL_0321) and the human embryonic kidney cell line HEK293T (Cat#: H4‐1401, Saiye Biological Technology, Suzhou, China, RRID: CVCL_0063) were obtained from Saiye Biological Technology in September 2023. Both cell lines were authenticated by short tandem repeat (STR) locus analysis and tested negative for mycoplasma contamination. The HT22 and HEK‐293T cells were cultured in Dulbecco's Modified Eagle Medium (DMEM, high glucose, C11995500BT, Gibco, China) supplemented with 20% fetal bovine serum (FBS, FB15015, Clark, VA, USA) and 1% (v/v) penicillin‐streptomycin (P1400, Solarbio, Beijing, China). Cells were maintained in a humidified incubator at 37°C with 5% CO_2_.

### 4.13 Western Blot Analysis

Protein samples were prepared from hippocampal tissues or cultured cells as follows. Tissues were homogenized, and cells were lysed in RIPA lysis buffer (Cat#: R0020, Solarbio, Beijing, China) supplemented with 100 mM PMSF (P0100, Solarbio, Beijing, China) to prevent proteolysis. After ultrasonication, samples were incubated on ice for 1 h to ensure complete lysis. Lysates were clarified by centrifugation at 12 000×*g* for 20 min at 4°C, and the resulting supernatants were collected as total protein extracts. Protein concentrations were determined using a BCA Protein Assay Kit (Cat#: PC0020, Solarbio, Beijing, China), with absorbance measured on an Infinite F200 microplate reader (Cat#: TECAN, Männedorf, Switzerland). Equal amounts of protein (35 µg per lane) were mixed with 5×SDS loading buffer (Cat#: P0140, Solarbio, Beijing, China) and denatured by heat treatment. Proteins were separated by 10% SDS‐PAGE and then transferred onto polyvinylidene fluoride (PVDF) membranes. Membranes were blocked with 5% non‐fat milk in Tris‐buffered saline containing 0.1% Tween‐20 (TBST) for 1 h at room temperature to block non‐specific binding. Subsequently, membranes were incubated overnight at 4°C with primary antibodies. After three washes with TBST, membranes were incubated with species‐matched secondary antibodies for 2 h at room temperature. Immunoreactive bands were visualized using an Odyssey IR Fluorescence Imaging System (Cat#: 987–07708, Odyssey, LI‐COR Biosciences, Lincoln, NE, USA), and band intensities were quantified. Relative densitometric analysis was performed using Image Studio Ver 5.2 software (LI‐COR Biosciences), with β‐actin serving as the internal loading control. The antibodies used in this study are listed in Table S14.

### 4.14 SiRNA Transfection

All small interfering RNA (siRNA) oligonucleotides used in this study were supplied by GenePharma (Suzhou, China). HT22 cells were seeded into 6‐well plates and cultured until reaching 60%–70% confluency, a density optimized for transfection efficiency. SiRNA transfection was performed using the siRNA‐mate Plus Transfection Kit (siRNA/miRNA universal type, GenePharma, Suzhou, China) strictly according to the manufacturer's protocol. Transfection complexes were prepared as follows. First, 75 pmol of siRNA was diluted in 42.5 µL of the kit‐supplied buffer and mixed by gentle pipetting. Next, 15 µL of siRNA‐mate plus transfection reagent was added, and the mixture was gently pipetted to ensure uniform complex formation. The prepared complexes were then incubated at room temperature for 15 min to allow stable complex assembly.

Subsequently, the complexes were added dropwise to 6‐well plates containing 2 mL of serum‐containing complete culture medium per well, ensuring even distribution across the cell monolayer. The culture medium was not replaced during the first 24 h post‐transfection to avoid disrupting the transfection complexes and reducing transfection efficiency. Cells were harvested at the indicated time points for downstream analyses. qRT‐PCR was performed 24 h post‐transfection to assess target gene mRNA knockdown efficiency, whereas Western blot was conducted at 48 h post‐transfection to evaluate corresponding protein knockdown levels. The sequences of the siRNAs used in this study are listed in Table S15.

### 4.15 Quantitative Reverse Transcription‐Polymerase Chain Reaction (qRT‐PCR) Assay

Total RNA was extracted from samples using the Total RNApure Reagent Kit (Cat#: ZP401, ZOMANBIO, Beijing, China) strictly following the manufacturer's instructions. For cDNA synthesis, 1 µg of total RNA was reverse‐transcribed into cDNA (complementary DNA) using HiScript III RT SuperMix (Cat#: R323, Vazyme, Nanjing, China). The thermocycling conditions were as follows: initial denaturation at 42°C for 2 min, reverse transcription at 37°C for 15 min, and enzyme inactivation at 85°C for 5 s. qPCR was performed using ChamQ Universal SYBR qPCR Master Mix (Cat#: KT201, Vazyme, Nanjing, China) on a QuantStudio 6 Flex Real‐Time PCR System (Cat#: 4484642, Applied Biosystems, Shanghai, China). The qPCR cycling program was as follows: initial polymerase activation at 95°C for 15 min, followed by 40 cycles consisting of denaturation at 95°C for 10 s, annealing at 58°C for 30 s, and extension at 72°C for 25 s. β‐actin was used as the endogenous reference gene to normalize mRNA expression, whereas U6 served as the endogenous reference gene for miRNA expression. Relative expression was calculated using the 2^−∆∆Ct^ method. The primer sequences used in this study are provided in Table S16.

### 4.16 RNA Fluorescence In Situ Hybridization (FISH) Assay

Gm26550‐specific probes, as well as U6 and 18S reference probes and 18S reference probes, were purchased from Geneseed (Guangzhou, China). miR‐26a‐5p‐specific probe was also purchased from Geneseed (Guangzhou, China). HT22 cells were seeded into 12‐well plates at an appropriate density and cultured until reaching the desired confluency. Prior to fixation, cells were gently rinsed with PBS and then fixed in 4% paraformaldehyde (PFA) at room temperature for 10 min. RNA FISH was performed using the RNA fluorescence in situ hybridization (RNA FISH) kit (Cat#: D‐2922B, EXONBIO, Guangzhou, China) in strict accordance with the manufacturer's instructions. Briefly, hybridization was carried out in a humidified chamber at 37°C–42°C in the dark overnight with the aforementioned specific probes. U6 probes were used as nuclear localization controls, while 18S probes served as cytoplasmic controls. After hybridization, cell nuclei were counterstained with DAPI. All fluorescent images were captured using a confocal laser scanning microscope (Olympus FV1200, Tokyo, Japan) with consistent imaging parameters across all samples.

### 4.17 RNA Immunoprecipitation (RIP) Assay

RIP assays were performed using the PureBinding RNA Immunoprecipitation Kit (GENESEED, Guangzhou, China) in strict accordance with the manufacturer's instructions. To summarize, HT22 cells or mouse hippocampal tissues were lysed in lysis buffer supplemented with protease inhibitor cocktail and RNase inhibitor to preserve RNA‐protein complexes. The resulting whole‐cell lysates were divided into three aliquots: the Input group (positive control), the IP antibody group, and the IgG antibody group (negative control). Meanwhile, magnetic beads provided in the kit were pretreated and then conjugated with either the target IP antibody or control IgG antibody to form bead‐antibody complexes. Subsequently, the bead‐antibody complexes were co‐incubated with the corresponding cell lysate aliquots at 4°C overnight with gentle rotation to allow specific antigen capture, forming bead‐antibody‐antigen complexes. After incubation, the complexes were collected using a magnetic rack and washed extensively with the kit‐supplied wash buffer to remove non‐specific interactions. The RNA bound to the complexes was eluted, and residual DNA was digested using DNase I. Finally, total RNA was extracted from the eluate using TRIzol Reagent (Cat#: ZP401, ZOMANBIO, Beijing, China), and qRT‐PCR was performed to detect the enriched RNA targets.

### 4.18 RNA Pull‐Down Assay

RNA pull‐down assays were performed using the PureBinding RNA‐Protein pull‐down Kit (Cat#: P0202, GENESEED, Guangzhou, China) strictly following the manufacturer's instructions. Biotinylated Gm26550‐specific was synthesized by Geneseed (Guangzhou, China). Briefly, HT22 cells or mouse hippocampal tissues were harvested, lysed, and sonicated to generate whole‐cell lysates. Meanwhile, the biotinylated probes were incubated with streptavidin‐conjugated magnetic beads (Geneseed, Guangzhou, China) at 25°C for 2 h to prepare probe‐coated beads. Subsequently, the prepared cell lysates were co‐incubated with the probe‐coated beads at 4°C overnight with gentle rotation. After extensive washing with the kit‐supplied wash buffer to remove non‐specifically bound materials, the RNA‐protein complexes bound to the beads were eluted according to the manufacturer's protocol. Finally, Western blot analysis was conducted to detect the enrichment of AGO2 and KHSRP proteins in the eluted complexes.

### 4.19 Chromatin Immunoprecipitation (ChIP)‐qPCR Assay

ChIP assays were carried out with a ChIP Assay Kit (Cat#: 17–295, Merck Millipore, Darmstadt, Germany) following the manufacturer's standard protocol. Briefly, HT22 cells were fixed with 1% formaldehyde at room temperature for 10 min for protein‐DNA cross‐linking, and the reaction was quenched by 125 mM glycine incubation for 5 min. Cells were washed twice with pre‐cooled PBS containing 1% protease inhibitor cocktail, harvested, and subjected to sonication to obtain fragmented cell lysates. For immunoprecipitation, lysates were incubated overnight at 4°C with anti‐Nrf2 antibody (Cat#: HA723302, HUABIO, Hangzhou, China), while rabbit IgG was applied as a negative control. Subsequent qPCR quantification was performed to detect Nrf2 enrichment at target regions. We selected the Nqo1 promoter as a well‐established positive control and an intergenic non‐binding genomic region (Neg) as an additional negative control to validate the reliability of the ChIP system. The full promoter sequence of Gm26550 was retrieved from the UCSC Genome Browser (https://genome.ucsc.edu/). Potential Nrf2‐binding motifs within this promoter region were predicted via the JASPAR database (https://jaspar.elixir.no/). Binding sites with a prediction score above 10 were retained, redundant sequences were eliminated, and the motif located at 1923–1937 bp of the Gm26550 promoter was selected for subsequent ChIP‐qPCR verification. All primer sequences used for ChIP‐qPCR are listed inTable S17.

### 4.20 Dual‐Luciferase Reporter Assay

The pEZX‐Gm26550 promoter vector, Nrf2 overexpression vector, and their corresponding control vectors (all constructed by GENERAL BIOL, Anhui, China) were co‐transfected into HEK‐293T cells using Lipofectamine 3000 (Cat#: L3000015, Thermo Fisher Scientific, Waltham, MA, USA), and incubated at 37°C in a humidified 5% CO_2_ incubator for 24 h. Subsequently, luciferase activity was determined using the Dual‐Luciferase Reporter System kit (Cat#: E2920, Promega Corporation) in strict accordance with the manufacturer's instructions. Relative fluorescence activity was calculated as the ratio of firefly luciferase fluorescence to Renilla luciferase fluorescence intensity.

Luciferase reporter vectors containing wild‐type Gm26550 (Gm26550‐WT), mutant Gm26550 (Gm26550‐MUT), wild‐type IGF1 3′‐untranslated region (IGF1 3′UTR‐WT) or mutant IGF1 (IGF1 3′UTR‐MUT) were constructed by GENERAL BIOL (Anhui, China). Subsequently, HEK‐293T cells were co‐transfected with the aforementioned reporter vectors and either miR‐26a‐5p mimics or NC mimics using Lipofectamine 3000. After transfection, cells were incubated under the same culture conditions for 24 h. Luciferase activity was then determined using the same Dual‐Luciferase Reporter Assay System kit (Cat#: E2920, Promega Corporation) following the manufacturer's protocol. Relative luciferase activity was calculated as the ratio of Renilla luciferase fluorescence intensity to firefly luciferase fluorescence intensity. The sequences of the wild‐type and mutant miR‐26a‐5p binding fragments inserted into the psiCHECK‐2 dual‐luciferase reporter vector used in this study are listed in Table S18.

### 4.21 Plasmids Transfection

All plasmids used in this study were generated by WZ Biosciences Inc. (Shandong, China). Plasmid isolation was performed as follows. *Escherichia coli* strains harboring the target plasmids were cultured in Luria‐Bertani (LB) medium supplemented with appropriate selective antibiotics. Plasmids were purified using the EasyPure HiPure Plasmid MiniPrep Kit (TRAN, Beijing, China) according to the manufacturer's instructions.

HT22 cells were seeded into 6‐well plates and cultured until reaching 60%–70% confluency, a density previously optimized for maximizing transfection efficiency. Plasmid transfection was performed using Lipofectamine 3000 (Cat#: L3000015, Thermo Fisher Scientific, Waltham, MA, USA) according to the manufacturer's instructions. Purified plasmids were mixed with Lipofectamine 3000 in Opti‐MEM reduced‐serum medium (Thermo Fisher Scientific, Waltham, MA, USA) to form transfection complexes, which were then incubated at room temperature for 15 min to ensure stable complex formation. The transfection complexes were subsequently added dropwise to the cell culture wells to ensure uniform distribution. The culture medium was not replaced within 24 h after transfection to avoid disrupting the transfection complexes and reducing transfection efficiency. Cells were harvested at predetermined time points post‐transfection for downstream analyses. qRT‐PCR was performed at 24 h post‐transfection to assess target gene mRNA expression, whereas Western blot was conducted at 48 h post‐transfection to evaluate corresponding protein expression levels. The RNA sequences of wild‐type Gm26550 and the full‐site mutants of Gm26550 with mutated binding regions used in this study are listed in Supporting Information Table S19.

### 4.22 Statistical Analysis

Statistical analyses were performed using GraphPad Prism 10.0 (GraphPad Software, CA, USA), and data are presented as mean ± standard error of the mean (SEM). Data normality and homogeneity of variance were assessed using the Shapiro–Wilk test and Levene's test, respectively. When data deviated from a normal distribution, log transformation was conducted; if normality and homogeneity of variance could not be restored after transformation, nonparametric statistical tests were adopted. Comparisons between two groups were conducted with the unpaired Student's t‐test for normally distributed data or the Mann–Whitney U test for non‐normal data. Comparisons across three or more groups were analyzed via one‐way or two‐way analysis of variance (ANOVA), followed by Bonferroni's post hoc multiple comparison tests. For clarity of sample size definition across all figures: For in vivo animal assays, n denotes the number of independent mice per experimental group; for in vitro cell‐based experiments, n denotes the number of independent biological samples. All experiments in this study were performed with three independent biological replicates. Statistical significance was defined as *p* < 0.05.

## Author Contributions

**Hongfang Wang**: writing – original draft, visualization, validation, methodology, investigation, formal analysis, data curation, and conceptualization. **Ziyao Wang and Dongyue Zuo**: writing – review & editing, supervision, methodology, and conceptualization. **Zhaowen Su**: visualization, methodology, investigation, formal analysis, data curation, and conceptualization. **Bowen Song and Jiamin Gao**: visualization, formal analysis, and data curation. **Yizhou Zhang**: validation, formal analysis, and funding acquisition. **Ruiting Zhao and Linjie Sun**: validation and formal analysis. **Mengdi Li and Yirui Fan**: visualization, formal analysis, and data curation. **Dandan Geng and Lei Wang**: writing – review & editing, supervision, resources, project administration, and funding acquisition.

## Conflicts of Interest

The authors declare no conflicts of interest.

## Supporting information




**Supporting File**: advs779599‐sup‐0001‐SuppMat.docx.

## Data Availability

The data that support the findings of this study are available from the corresponding author upon reasonable request.
