# Supplementary material for: Gm26550 Modulates Learning and Memory by Increasing IGF1 mRNA Expression and Stability in Nrf2−/− Mice
Source: Adv Sci (Weinh). 2026 Sep 27:e24087. Online ahead of print. doi: 10.1002/advs.202524087 (PMC13616242; doi:10.1002/advs.202524087)
Supplement: Supplementary file 1 [file ADVS-9999-e24087-s001.docx]

**Supporting Information**

**Gm26550 Modulates Learning and Memory by Increasing IGF1 mRNA Expression and Stability in Nrf2^-/-^ Mice**

***Hongfang Wang^1,^****^†^****, Ziyao Wang^2,^****^†^****, Dongyue Zuo^1,^****^†^****, Zhaowen Su^1^, Bowen Song^3^, Jiamin Gao^1^, Yizhou Zhang^1,4,5^, Ruiting Zhao^1^, Linjie Sun^1^, Mengdi Li^6^, Yirui Fan^6^, Dandan Geng^1,4,5*^, and Lei Wang^1,4,5,7*^***

1 Department of Human Anatomy, Hebei Medical University, Shijiazhuang 050017, China.

2 Grade 2023, 5+3 Clinical Medicine, Hebei Medical University, Shijiazhuang 050017, China.

3 Grade 2023, Clinical Medicine, Hebei Medical University, Shijiazhuang 050017, China.

4 Hebei Key Laboratory of Neurodegenerative Disease Mechanism, Shijiazhuang 050017, China.

5 Neuroscience Research Center, Hebei Medical University, Shijiazhuang 050017, China.

6 Grade 2023, Clinical Medicine, Clinical College of Hebei Medical University, Shijiazhuang 050017, China.

7 The Key Laboratory of Neural and Vascular Biology, Ministry of Education, Hebei Medical University, Shijiazhuang 050017, China.

***Correspondence:**

gengdandan@hebmu.edu.cn (Dandan Geng); wanglei@hebmu.edu.cn (Lei Wang).

**† These authors contributed equally to this work.**

**Supplementary Figures**

Figure S1. The expression of Gm26550 is down-regulated in the hippocampus of Nrf2^-/-^ mice.

(A) Agarose gel electrophoresis of PCR products and genotyping analysis of Nrf2^-/-^ mice. (n = 4). (B) qRT-PCR analysis of Nrf2 mRNA in the hippocampus of Nrf2^-/-^ mice. (n = 4). (C-E) The NOR test was performed at 2 h and 24 h to assess the preference of mice for familiar and novel objects. (n = 15). (F-J) The MWM test was performed to evaluate spatial learning and memory. (n = 15). (F) Escape latency time (average latency) from Days 2 to 6. (G-H) Time spent in the target quadrant and the number of platform crossings on Day 7. (I) Average velocity during the probe trial. (J) Representative swimming trajectories from the two groups on Day 7. (K-L) Sequence conservation of the Gm26550 gene locus among different species. (M) qRT-PCR was used to assess the efficiency of Nrf2 knockdown. (n = 4). (N-O) Western blot was used to assess the efficiency of Nrf2 knockdown. (n = 4). (P-R) qRT-PCR and Western blot were used to assess the efficiency of Nrf2 overexpression. (n = 4). (S) Nrf2 binding motifs and corresponding sequences were predicted using the JASPAR database. Data are presented as the mean ± SEM. **P* < 0.05, ***P* < 0.01, and ****P* < 0.001 by two-tailed t test (B, D, E, G-I, O, P, and R) or two-way ANOVA (F) or one-way ANOVA (M) followed by Bonferroni’s post hoc test.

Figure S2. Gm26550 is decreased in 5×FAD mice and AD patients.

(A) Gm26550 expression in the hippocampus of 5×FAD mice versus WT controls. (n = 3). (B) Gm26550 expression in AD patient hippocampus (n = 5) versus controls (n = 6). Data are presented as the mean ± SEM. **P* < 0.05, ***P* < 0.01 by two-tailed t test (A and B).

Figure S3. Knockdown of Gm26550 decreases PSD95 and SYN1 expression in HT22 cells. (A) Representative Western blot images of PSD95 and SYN1 expression in HT22 cells. (n = 4). (B-C) Quantification of PSD95 and SYN1 expression. Data are presented as the mean ± SEM. ***P* < 0.01 by two-tailed t test (B and C).

Figure S4. Co-expression analysis of Gm26550 and synaptic mRNAs in mouse hippocampus.

(A) Co-expression network centered on hub lncRNA Gm26550. Red edges indicate positive co-expression relationships, whereas blue edges indicate negative co-expression. The orange rectangle node indicates Gm26550; red circular nodes denote mRNAs up-regulated in Nrf2 knockout mice, and light blue circular nodes denote mRNAs down-regulated in Nrf2 knockout mice. (B) Summary table of Pearson and Spearman correlation coefficients, original P-values, and bootstrap 95% confidence intervals for all significantly co-expressed mRNA partners of Gm26550. (C) Linear regression scatter plot showing Pearson correlation between Gm26550 and IGF1 expression across six hippocampal samples. The grey shaded area denotes the bootstrap 95% confidence band; Pearson's r and its 95% CI are shown in the top-left corner. (D) Histogram of 2000 bootstrap resampling replicates for the Pearson correlation coefficient between Gm26550 and IGF1. The black dashed line marks the original Pearson r value, and blue dotted lines denote the lower and upper bounds of the bootstrap 95% confidence interval. (E) Scatter plot displaying Spearman rank correlation between Gm26550 and IGF1 expression in six hippocampal samples, with Spearman’s r and its bootstrap 95% CI annotated. (F) Histogram of 2000 bootstrap resampling replicates for the Spearman correlation coefficient between Gm26550 and IGF1. The black dashed line represents the original Spearman r value.

Figure S5. Verification of Gm26550 knockdown and overexpression efficiency.

(A) qRT-PCR was used to assess the efficiency of Gm26550 knockdown. (n = 5). (B) The expression level of Gm26550 in HT22 cells was measured by qRT-PCR following Gm26550 overexpression. (n = 5). Data are presented as the mean ± SEM. ***P* < 0.01, ****P* < 0.001 by one-way ANOVA followed by Bonferroni’s post hoc test (A) or two-tailed t test (B).

Figure S6. Gm26550 acts as a miRNA sponge.

(A) Schematic diagram exhibiting the overlap of miRNA targets of Gm26550 predicted by miRanda, TargetScan, miRcode, and RNAhybrid. (B) RIP assays were performed in HT22 cells using an anti-AGO2 antibody. Western blot was then used to detect AGO2 enrichment in the immunoprecipitated complexes. (C) Gm26550 RNA pull-down assays were performed in HT22 cells, followed by Western blot analysis to detect AGO2 enrichment in the pulled-down complexes.

Figure S7. Gm26550 interacts with KHSRP protein.

(A-B) Venn diagrams showing the overlap of potential RBPs interacting with Gm26550, as predicted by RBPmap, RBPDB, catRAPID, and RPISeq. (C) The RPISeq database predicts the interaction probability between Gm26550 and KHSRP. (D) PPI network of genes enriched by STRING. (E-F) RIP-qRT-PCR analysis was performed in HT22 cells to detect the enrichment of Gm26550 in KHSRP immunoprecipitated complexes. (n = 4). (G) Agarose gel electrophoresis was performed to verify the qRT-PCR products of Gm26550 enriched in KHSRP immunoprecipitates from hippocampal tissues of WT mice. (H) Gm26550 RNA pull-down was performed in HT22 cells, followed by Western blot to detect KHSRP enrichment in the pulled-down complexes. (n = 3). (I-K) KHSRP expression levels in HT22 cells were assessed by qRT-PCR and Western blot following Gm26550 knockdown. (n = 5). (L-N) KHSRP expression levels in HT22 cells were assessed by qRT-PCR and Western blot following Gm26550 overexpression (n = 5). Data are presented as the mean ± SEM. *P* > 0.05, ***P* < 0.01 by two-tailed t test (E, I, K, L, and N).

Figure S8. IGF1 mRNA associates with KHSRP through AREs.

(A) The KHSRP binding sites in the IGF1 3´UTR predicted by RBPmap. The bottom yellow vertical lines represent the KHSRP binding sites in the 3'UTR of IGF1 mRNA. (B) The motif of KHSRP. (C) The 3´UTR of IGF1 mRNA (highlighted in red) contains 60% AU residues. (D) qRT-PCR was used to assess the efficiency of KHSRP knockdown. (n = 5). (E) qRT-PCR was used to assess the efficiency of KHSRP overexpression. (n = 5). (F) qRT-PCR was used to measure IGF1 mRNA levels over time in HT22 cells following KHSRP knockdown. (n = 5). (G) qRT-PCR was used to measure IGF1 mRNA levels over time in HT22 cells following KHSRP overexpression. (n = 3). (H-J) IGF1 expression in HT22 cells was analyzed by qRT-PCR and Western blot following KHSRP overexpression. (n = 5). (K) qRT-PCR was used to assess IGF1 mRNA levels over time in HT22 cells after cotransfection with si-Gm26550 and si-KHSRP. (n = 4). (L) RIP assays were performed in HT22 cells using an anti-KHSRP antibody, and IGF1 mRNA enrichment in the immunoprecipitated complexes was determined by qRT-PCR. (n = 4). (M) RBPmap predicted a cluster of potential KHSRP binding sites within the IGF1 3′UTR. Data are presented as the mean ± SEM. **P* < 0.05, ***P* < 0.01, ****P* < 0.001, **^###^***P* < 0.001 by one-way ANOVA (D, F, and K) or two-way ANOVA (G) followed by Bonferroni’s post hoc test or two-tailed t test (E, H, J, and L).

Figure S9. The 359-410 nt of Gm26550 interact with the KH3/KH4 domains of KHSRP.

(A-C) The catRAPID database predicts the interaction sites between Gm26550 and KHSRP. (D) The MEME database predicts the interaction probability between Gm26550 and KHSRP. (E) Representative immunoblots demonstrating the products of FLAG-mediated immunoprecipitation in HEK293 cells expressing KHSRP-FLAG and Gm26550 fragments. IgG was used as the control. (F) Representative immunoblots demonstrating the products of FLAG-mediated immunoprecipitation in HEK293 cells expressing KH1-4-GFP-FLAG fragments and Gm26550. IgG was used as the control. (G) Representative immunoblots demonstrating the products of FLAG-mediated immunoprecipitation in HEK293 cells expressing KH1-4-GFP-FLAG fragments and IGF1 mRNA. IgG was used as the control. (H) Gm26550 and IGF1 mRNA enrichment were assessed by KHSRP-mediated pull-down followed by qRT-PCR. (n = 3). (I) Agarose gel electrophoresis was performed to verify qRT-PCR products. Data are presented as the mean ± SEM. **P* < 0.05, ****P* < 0.001 by two-way ANOVA (H) followed by Bonferroni’s post hoc test.

Figure S10. Schematic map and predicted secondary structures of Gm26550 wild-type and mutant variants.

(A) Schematic representation of the full-length Gm26550 sequence (1184 nt). The KHSRP-binding region (359-410 nt) and the miR-26a-5p binding site (1026-1051 nt) are indicated by pink and orange boxes, with their corresponding nucleotide sequences shown below. (B) Predicted minimum free energy (MFE) secondary structure of wild-type Gm26550 (Gm26550-WT) generated by RNAfold. Red circles highlight the two spatially distinct functional modules. (C) Predicted secondary structure of the KHSRP-binding region deletion mutant (Mut-Δ359-410). After removing 52 nt, the miR-26a-5p binding site shifts to nt 974-999. The red circle indicates that the local secondary structure of this region remains unchanged. (D) Predicted secondary structure of the miR-26a-5p binding site mutant (Mut-miR). The red circle highlights the mutated region. (E) Predicted secondary structure of the double mutant (Mut-miR+Δ359-410). The red circle indicates the mutated miRNA binding site within the KHSRP-deleted background.

**Supplementary Tables**

**Table S1. Significantly upregulated lncRNAs in the hippocampus of Nrf2^-/-^ versus WT mice**

**Table S2. Significantly downregulated lncRNAs in the hippocampus of Nrf2^-/-^ versus WT mice**

**Table S3. Non-redundant set of significantly upregulated lncRNAs in the hippocampus of Nrf2^-/-^ versus WT mice**

**Table S4. Non-redundant set of significantly downregulated lncRNAs in the hippocampus of Nrf2^-/-^ versus WT mice**

**Table S5. Significantly upregulated mRNAs in the hippocampus of Nrf2^-/-^ versus WT mice**

**Table S6. Significantly downregulated mRNAs in the hippocampus of Nrf2^-/-^ versus WT mice**

**Table S7. Non-redundant set of significantly upregulated mRNAs in the hippocampus of Nrf2^-/-^ versus WT mice**

**Table S8. Non-redundant set of significantly downregulated mRNAs in the hippocampus of Nrf2^-/-^ versus WT mice**

**Table S9. Orthologous human HGNC-matched gene list of differentially expressed genes from Nrf2^-/-^ mice**

**Table S10. Overlap of SynGO 1.3 Synaptic Genes and Nrf2^-/-^ DEG Orthologs**

**Table S11. Pearson correlation coefficients and 95% confidence intervals between Gm26550 and co-expressed mRNAs in the hippocampus of Nrf2^-/-^ versus WT mice**

**Table S12. Spearman correlation coefficients and 95% confidence intervals between Gm26550 and co-expressed mRNAs in the hippocampus of Nrf2^-/-^ versus WT mice**

**Table S13. The information of brain samples of normal and patients with AD.**

| **Type** | **Gender** | **Age** | **Tissue** |
| --- | --- | --- | --- |
| Control | Male | 70 | Hippocampus |
| Control | Male | 52 | Hippocampus |
| Control | Male | 61 | Hippocampus |
| Control | Male | 90 | Hippocampus |
| Control | Male | 66 | Hippocampus |
| Control | Female | 77 | Hippocampus |
| AD patient | Female | 92 | Hippocampus |
| AD patient | Male | 84 | Hippocampus |
| AD patient | Female | 88 | Hippocampus |
| AD patient | Male | 80 | Hippocampus |
| AD patient | Female | 92 | Hippocampus |

**Table S14. Antibodies used in this study.**

| **Antibody** | **Source** | **Identifier** |
| --- | --- | --- |
| Rabbit anti-Nrf2 | Proteintech | Cat#:66504-1-Ig; RRID:AB_2881867 |
| Rabbit anti-Nrf2 | HUABIO | Cat#: HA751401; RRID: N/A |
| Rabbit anti-β-Actin | ABclonal | Cat#: AC026; RRID: AB_2768234 |
| Rabbit anti-PSD95 | Cohesion | Cat#: CMA4405; RRID: N/A |
| Rabbit anti-SYN1 | Cohesion | Cat#: CPA7127; RRID: N/A |
| Rabbit anti-IGF1 | Cohesion | Cat#: CPA5435; RRID: N/A |
| Mouse anti-IGF1 | OriGene | Cat#: TA805748S; RRID: AB_2627844 |
| Rabbit anti-KHSRP | Proteintech | Cat#:55409-1-AP; RRID:AB_11182170 |
| Rabbit anti-AGO2 | ABclonal | Cat#: A6802; RRID: AB_2767383 |
| Mouse anti-AGO2 | Proteintech | Cat#: 67934-1-Ig; RRID:AB_2918686 |
| Rabbit anti-PABPC1 | Proteintech | Cat#:10970-1-AP; RRID:AB_10596918 |
| Rabbit anti-KHDRBS3 | Proteintech | Cat#:13563-1-AP; RRID:AB_2130319 |
| Rabbit anti-MBNL1 | Proteintech | Cat#:66837-1-Ig; RRID:AB_2882180 |
| Mouse anti-FLAG | Abmart | Cat#: M20008; RRID: AB_2713960 |
| Rabbit anti-IgG | Proteintech | Cat#:30000-0-AP; RRID:AB_2819035 |
| Mouse anti-IgG | ABclonal | Cat#: AC011; RRID: AB_2770414 |
| Rabbit IgG (H&L) Antibody DyLight 800 Conjugated | Rockland | Cat#:611-145-002; RRID:AB_1660964 |
| [Mouse IgG (H&L) Antibody DyLight 549 Conjugated](https://rrid.site/data/record/nif-0000-07730-1/RRID:AB_11182796/resolver?q=610-142-002&i=rrid:ab_11182796-1246450) | Rockland | Cat#:610-130-121; RRID: AB_11182796 |

**Table S15. Sequences of siRNA used in this study.**

| **SiRNA name** | **Sequence** |
| --- | --- |
| Negative control | Forward: UUCUCCGAACGUGUCACGUTT |
|  | Reverse: ACGUGACACGUUCGGAGAATT |
| Si-Nrf2-1 | Forward: AAAGCUAGUAUAGCAAUAATT |
|  | Reverse: UUAUUGCUAUACUAGCUUUGG |
| Si-Nrf2-2 | Forward: UGAAGUCUUCAGCAUGUUATT |
|  | Reverse: UAACAUGCUGAAGACUUCAAG |
| Si-Gm26550-1 | Forward: GCAUGAUGUAAGCCCAUAATT |
|  | Reverse: UUAUGGGCUUACAUCAUGCTT |
| Si-Gm26550-2 | Forward: GGACCUUCGAGAUUGAUUATT |
|  | Reverse: UAAUCAAUCUCGAAGGUCCTT |
| Si-KHSRP-1 | Forward: GAGGUGGUGAGCAGAUAAATT |
|  | Reverse: UUUAUCUGCUCACCACCUCTT |
| Si-KHSRP-2 | Forward: GGGAGGAGUACUACAAGAATT |
|  | Reverse: UUCUUGUAGUACUCCUCCCTT |
| miR-26a-5p mimics | Forward: UUCAAGUAAUCCAGGAUAGGCU |
|  | Reverse: CCUAUCCUGGAUUACUUGAAUU |
| miR-26a-5p inhibitor | Reverse: AGCCUAUCCUGGAUUACUUGAA |

**Table S16. qRT-PCR primer sequences used in this study.**

| **Primer name** | **Sequence** |
| --- | --- |
| m Nrf2 | Reverse: AAAATCATTAACCTCCCTGTTGAT |
|  | Forward: CGGCGACTTTATTCTTACCTCTC |
| m Gm26550 | Forward: ACTGTCTTCATCCTGTGCGG |
|  | Reverse: CTGTGTGAAGGTGGGTGTGT |
| m KHSRP | Forward: TTCCGAAGCTCTGACTGGTC |
|  | Reverse: AAGGGTCCAGGGTTGAAAGG |
| m IGF1 | Forward: CCGAGGGGCTTTTACTTCAAC |
|  | Reverse: CAGTCTCCTCAGATCACAGCT |
| m β-Actin | Forward: TCATCACTATTGGCAACGAGCGGT |
|  | Reverse: GTGTTGGCATAGAGGTCTTTACG |
| h Gm26550 (NONHSAT179774) | Forward: GATGAGTAACCTCGCCCCAG |
|  | Reverse: GGTGACTGAAGTGCTCGTCA |
| h β-Actin | Forward: GGCACCCAGCACAATGAAG |
|  | Reverse: CCGATCCACACGGAGTACTTG |

**Table S17. ChIP-qPCR primer sequences used in this study.**

| **Primer name** | **Sequence** |
| --- | --- |
| Gm26550 Promoter | Forward: CAATCCCGGCTACTGAGGAG |
|  | Reverse: ACGTTCAGACAGGCCCTTTT |
| NQO1 Promoter | Forward: GCACGAATTCATTTCACACGAGG |
|  | Reverse: GGAAGTCACCTTTGCACGCTAG |
| Negative control | Forward: GCCACCAGGTGACTCAGGAA |
|  | Reverse: TCCTTTCAGCTTCCTGAGTCAC |

**Table S18. Sequences of wild-type and mutant miR-26a-5p binding fragments inserted into psiCHECK-2 dual-luciferase reporter vectors used in this study.**

| **name** | **Sequence** |
| --- | --- |
| psiCHECK-2-Gm26550-WT  (1026-1051 nt) | ACCUGUCAUCCAGGGAAGACUCAGAG |
| psiCHECK-2-Gm26550-Mut  (1026-1051 nt) | **G**C**UC**G**C**C**GCUU**AG**AAG**AG**GUC**CA**AGA** |

**Table S19. RNA sequences of wild-type Gm26550 and full-site mutant Gm26550 with mutagenized binding region used in this study.**

| **name** | **Sequence** |
| --- | --- |
| pcDNA3.1-Gm26550-WT  (1-1184 nt) | 5'-GACT…GGUGGGGUCACCUGUCAUCCAGGGAAGACUCAGAGGGAGAAAGUG…AAGG-3' |
| pcDNA3.1-Gm26550-mut-miR  (1-1184 nt) | 5'-GACT…GGUGGGGUC**G**C**UC**G**C**C**GCUU**AG**AAG**AG**GUC**CA**AGA**GGAGAAAGUG…AAGG-3' |
